# Maternal Immune Activation Induces Cortical Catecholaminergic Hypofunction and Cognitive Impairments in Offspring

**DOI:** 10.1007/s11481-023-10070-1

**Published:** 2023-05-20

**Authors:** Blanca Perez-Palomar, Amaia M. Erdozain, Ines Erkizia-Santamaría, Jorge E. Ortega, J. Javier Meana

**Affiliations:** 1 Department of Pharmacology, University of the Basque Country UPV/EHU, Leioa, Bizkaia, E-48940 Spain; 2 Centro de Investigación Biomédica en Red de Salud Mental CIBERSAM, ISCIII, Leioa, Spain; 3 Biocruces Bizkaia Health Research Institute, Bizkaia, Spain; 4 Department of Anesthesiology, Washington University in St. Louis, St. Louis, MO 63110 USA; 5 Department of Pharmaceutical and Administrative Sciences, University of Health Sciences and Pharmacy in St. Louis, St. Louis, MO 63110 USA

**Keywords:** Maternal Immune Activation, Microdialysis, Dopamine, Noradrenaline, D_1_ Receptors, D_2_ Receptors, Cognitive Impairment

## Abstract

**Background:**

Impairment of specific cognitive domains in schizophrenia has been associated with prefrontal cortex (PFC) catecholaminergic deficits. Among other factors, prenatal exposure to infections represents an environmental risk factor for schizophrenia development in adulthood. However, it remains largely unknown whether the prenatal infection-induced changes in the brain may be associated with concrete switches in a particular neurochemical circuit, and therefore, if they could alter behavioral functions.

**Methods:**

In vitro and in vivo neurochemical evaluation of the PFC catecholaminergic systems was performed in offspring from mice undergoing maternal immune activation (MIA). The cognitive status was also evaluated. Prenatal viral infection was mimicked by polyriboinosinic-polyribocytidylic acid (poly(I:C)) administration to pregnant dams (7.5 mg/kg i.p., gestational day 9.5) and consequences were evaluated in adult offspring.

**Results:**

MIA-treated offspring showed disrupted recognition memory in the novel object recognition task (*t* = 2.30, *p* = 0.031). This poly(I:C)-based group displayed decreased extracellular dopamine (DA) concentrations compared to controls (*t* = 3.17, *p* = 0.0068). Potassium-evoked release of DA and noradrenaline (NA) were impaired in the poly(I:C) group (DA: *F*_*t*_[10,90] = 43.33, *p* < 0.0001; *F*_*tr*_[1,90] = 1.224, *p* = 0.2972; *F*_*i*_[10,90] = 5.916, *p* < 0.0001; n = 11); (NA: *F*_*t*_[10,90] = 36.27, *p* < 0.0001; *F*_*tr*_[1,90] = 1.841, *p* = 0.208; *F*_*i*_[10,90] = 8.686, *p* < 0.0001; n = 11). In the same way, amphetamine‐evoked release of DA and NA were also impaired in the poly(I:C) group (DA: *F*_*t*_[8,328] = 22.01, *p* < 0.0001; F_tr_[1,328] = 4.507, *p* = 0.040; F_i_[8,328] = 2.319, *p* = 0.020; n = 43); (NA: F_t_[8,328] = 52.07; *p* < 0.0001; F_tr_[1,328] = 4.322; *p* = 0.044; F_i_[8,398] = 5.727; *p* < 0.0001; n = 43). This catecholamine imbalance was accompanied by increased dopamine D_1_ and D_2_ receptor expression (*t* = 2.64, *p* = 0.011 and *t* = 3.55, *p* = 0.0009; respectively), whereas tyrosine hydroxylase, DA and NA tissue content, DA and NA transporter (DAT/NET) expression and function were unaltered.

**Conclusions:**

MIA induces in offspring a presynaptic catecholaminergic hypofunction in PFC with cognitive impairment. This poly(I:C)-based model reproduces catecholamine phenotypes reported in schizophrenia and represents an opportunity for the study of cognitive impairment associated to this disorder.

**Supplementary Information:**

The online version contains supplementary material available at 10.1007/s11481-023-10070-1.

## Background

Schizophrenia is conceptualized as a clinical syndrome with core features clustered into positive symptoms (i.e. hallucinations and delusions), negative symptoms (i.e. social withdrawal and lack of motivation) and cognitive deficits (i.e. disturbances in selective attention, working memory and learning). Although psychosis identifies schizophrenia, decline in cognitive functioning precedes the onset of schizophrenia and remains relatively stable over the course of the illness, representing the strongest factor of functional outcome for individuals with schizophrenia [1]. The prefrontal cortex (PFC) is the main anatomical substrate of cognitive activities, particularly working memory, being the latter a cardinal component of executive functions [2–5]. It is well known that the different monoamines modulate and exert complementary functions in the PFC. Indeed, extracellular concentrations of dopamine (DA) and noradrenaline (NA) in the area act in concert and may rapidly alter the strength of the PFC network connections to coordinate cognitive status with physiological demands [4, 6, 7].

Neuropathological and neuroimaging studies have shown molecular and anatomical alterations in the PFC of schizophrenia patients [8]. Classical studies suggest that an excess in DA release might cause schizophrenia symptoms (for review, see McCutcheon et al. [9]). Currently, the DA imbalance hypothesis of schizophrenia proposes that enhanced activity in the mesolimbic dopaminergic system together with hypoactive mesocortical DA projections to the PFC contribute to the pathophysiology of the disease [10, 11]. Accordingly, different results show correlation between the positive psychotic symptoms and an increase of DA release in the striatum [12]. On the contrary, negative and cognitive dysfunctions in schizophrenia patients are associated to functional DA deficits in PFC [13–16]. Recently, a deficit in cortical DA transmission has been shown to correlate with increased striatal DA in patients with schizophrenia [15].

Evidence states that disruption of early brain development by a combination of genetic and environmental factors increases the risk of developing schizophrenia later in adulthood [17–19]. Especially affected processes include local circuitry formation and maturation, comprising cell proliferation, migration, arborization, myelination, connectivity and activity-dependent maturation [20, 21]. All these changes would merge revealing a network disruption, and eventually, cognitive impairment. However, it is not yet fully understood how these mechanisms trigger upstream and/or downstream changes in the different neuronal circuits that ultimately lead to behavioral dysfunction.

Among environmental factors, increasing evidence points out maternal exposure to a variety of viral, bacterial, and protozoan infections during pregnancy as relevant environmental risk factors of neurodevelopmental brain dysfunctions in the offspring [22–29]. These findings have led to the hypothesis that the infection acts as a disease primer, producing the release of common inflammatory mediators that are responsible for the divergence of natural fetal brain development after maternal infection [30, 31]. Still, it remains largely unknown whether the prenatal infection-induced changes in the brain may be associated with concrete switches in a particular neurochemical circuit, and therefore, if they could alter behavioral functions. In this context, animal models have been fundamental to provide insight regarding the neurobiological mechanisms of human brain disorders, including schizophrenia. Based on the construct premises described above, rodent and, more recently, nonhuman primate maternal immune activation (MIA) models have been developed. They provided substantial evidence of a causal relationship between prenatal exposure to infectious and/or immune activating agents and the emergence of brain dysfunctions in the adult life [31–38]. In utero, viral-like immune activation is commonly modeled by injecting polyriboinosinic-polyribocytidylic acid (poly(I:C)) to pregnant dams. Poly(I:C) is a synthetic double-stranded ribonucleic acid (dsRNA) whose action is based on the activation of the toll-like receptor 3 (TLR3), a member of the innate immune system’s pattern recognition receptors TLR family. Upon binding to the TLR3, poly(I:C) can efficiently mimic the acute inflammatory phase response to viral infection, stimulating the production and release of many pro-inflammatory cytokines [31, 39–42]. Under such circumstances, the integrity of the placental barrier becomes compromised, allowing the entrance of maternally derived cytokines into the fetal circulation and inducing inflammatory responses in the developing fetus, including the brain [31]. This leads to structural and developmental disturbances in the model, which eventually emerge as schizophrenia-associated phenotypes [28, 35, 43]. Accordingly, an important study in non-human primates has recently demonstrated that prenatal poly(I:C) administration alters brain growth leading to smaller frontal grey and white matter volume and changes in cognitive development [38].

Although catecholaminergic neurons and their target regions play essential roles in the pathophysiology of schizophrenia, it remains to be elucidated whether DA and NA functionality in the PFC is disrupted in MIA models, and if such alterations could be associated with cognitive impairment in offspring. In this context, this work aimed to perform a behavioral and neurochemical characterization of the poly(I:C) MIA model in mice, particularly emphasizing its potential use as an animal model of the cognitive impairment associated with schizophrenia (CIAS). With that purpose, an in vivo neurochemical evaluation of the catecholaminergic neurotransmission systems was carried out in the area by the microdialysis technique. In addition, in vitro studies were performed to determine the status of the different components of the dopaminergic and noradrenergic systems in the PFC. Moreover, behavioral studies were conducted in the MIA model to evaluate cognitive domains that have been shown as clearly impaired in schizophrenia patients.

## Materials and Methods

### Animals

For all the experiments and assays wild-type Hsd: ICR (CD1) adult male mice aged between eight and ten weeks were used. On a 12 h light/dark cycle, they were housed, five per cage, at room temperature (22–25 °C) and 65–70% humidity, with access to food and water *ad libitum*. Animals were obtained from the Animal Facility of the University of the Basque Country UPV/EHU.

Animal care and experimental protocols were executed following the principles of animal care established by the European Directive for the Protection of Vertebrate Animals used for experimental and other scientific purposes (European Union Directive 2010/63/UE) and by European Ethical Standards (6106/10-EEC), as translated to the Spanish legislation that regulates the welfare of animals used in experimentation and other scientific purposes (Royal Decree 53/2013). Besides, the Committee of Ethics for Animal Experimentation of the University of the Basque Country UPV/EHU approved all the experimental protocols. Experiments were conducted in accordance with the ARRIVE guidelines for animal studies.

### Breeding and Maternal Immune Activation Paradigm

Male CD1 mice were singly housed for three consecutive days. On the fourth day, two 10-week-old female mice were introduced to the age-matched males and left undisturbed for five days. Dams were checked daily for the presence of vaginal plug. If there was a vaginal plug, those females were separated from the male and housed in different cages. That day was recorded as gestational day (GD) 0.5. On GD 9.5, female mice received an intraperitoneal (i.p.) injection of 7.5 mg/kg of poly(I:C) (Batch: 095M4049V, Sigma-Aldrich, St. Louis, MO, USA) dissolved in 0.9% saline, or vehicle (saline 0.9%) (5 ml/kg). This period corresponds to approximately the middle/end of the first trimester of human pregnancy with respect to developmental biology and represents a critical moment of brain development in mouse gestation [44–46]. Doses above 5 mg/kg administered on GD 9.5 have previously shown to produce behavioral and neurochemical changes in mice offspring [47–52]. Then, dams were left undisturbed in groups of two animals and were supervised every day until the end of the pregnancy. Pups remained with their mothers until the weaning on postnatal day (PD) 21, and finally littermates were group-housed by sex (four-five per cage). In total, 106 mothers and 216 pups coming from different litters were used in this study. The number of animals included for each test is detailed in Additional file 1: Supplementary Table 1. All the experimental procedures were performed in adult descendant males of saline and poly(I:C)-treated mothers, which are termed along this text as saline and poly(I:C) groups, respectively. Each animal was exclusively used for one single experimental test. To minimize potential confounding factors, such as the order of treatments and measurements, all procedures were performed in completely randomized order. One experimenter performed the experiments and a different one was responsible for the blind analysis of the data. Following the guidelines for the responsible use of experimental animals, the females were assigned to additional projects.

### Drugs and Reagents

Alpha-methyl-phenethylamine (amphetamine), 2-(3,4-dihydroxyphenyl)ethylamine hydrochloride (DA), 5S,10R)-(+)-5-methyl-10,11-dihydro-5H-dibenzo [a,d] cyclohepten-5,10-imine hydrogen maleate or dizocilpine (MK-801), l-(−)-noradrenaline (+)-bitartrate salt monohydrate (NA), polyriboinosinic-polyribocytidylic acid (poly(I:C)), antipain, aprotinin, bromophenol blue, bovine albumin (BSA), β-mercaptoethanol, 1,4-dithiothreitol (DTT), glycerol, glycine, igepal, protease inhibitor cocktail, sodium dodecyl sulfate (SDS), sodium vanadate (Na_3_VO_4_), sodium floride (NaF), sodium azide (NaN_3_), sodium deoxycholate, Tris (2-amino-2-hydroxymethyl-1,3-propanediol) HCl, Triton™ X-100, sodium octyl sulfate and Tween 20 were obtained from Sigma Aldrich (St. Louis, MO, USA). Ammonium persulfate (APS), Bradford protein analysis method, N, N, N, N’-tetramethylethylelendiamine (TEMED), and Laemli buffer came from Bio-Rad Laboratories (Hercules, CA, USA). Acrylamide 30%-bysacrylamide 0.8% was obtained from National Diagnostics (Atlanta, GA, USA). The anesthetic gas 1-chloro-2,2,2-trifluoromethyl difluoromethyl ether (Isoflurane) was obtained from IsoFlo (Esteve Veterinaria, Barcelona, Spain) and the oxygen (O_2_) from Carburos Metálicos (Barcelona, Spain). Calcium chloride dihydrate (CaCl_2_), sodium chloride (NaCl), ethylenediaminetetraacetic acid, disodium salt dihydrate (EDTA), magnesium chloride (MgCl_2_), potassium chloride (KCl), sodium hydroxide (NaOH) were provided by Merck Millipore (Darmstadt, Germany). Citric acid monohydrate, 99.5% and 1-octane sulfonic acid High Performance Liquid Chromatography (HPLC) grade (OSA) were from Thermo Fisher Scientific (Waltham, MA, USA). Acetonitrile, glacial acetic acid and ortho-phosphoric acid 85% (H_3_PO_4_) were provided by Panreac Química S.A.U (Barcelona, Spain). Perchloric acid 60% was from Probus S.A. (Barcelona, Spain). Methanol was from Carlo Erba Reagents (Val de Reouil, France). All reagents were of the highest purity available in the standard commercial sources. Doses of the drugs are in reference to the salt forms.

**Behavioral assays: novel object recognition test (NORT**).

The spontaneous object recognition exploits the natural tendency of rodents to explore novel stimuli in preference to familiar stimuli. Recognition memory refers to the ability to judge a previous encountered item as familiar. The NORT is a behavioral test that uses novelty to evaluate time spent with a novel object versus a familiar object, as a measure of spatial and visual learning and memory evocation capacity [53]. This test consists of four consecutive days of experimental trials: a first day for habituation, two days of training and the last day for testing. In order to allow habituation to the new environment, mice were kept in the testing room for at least 30 min before the training and test sessions. Behavioral testing was performed between 9 a.m. and 12 p.m. On the first day, animals were habituated to the empty box (square-shaped wooden box 45 × 45 × 40 cm) and allowed to freely explore the arena for 10 min. In the next two days, animals were allowed to freely explore (10 min) two identical objects located in opposite corners of the arena. On the testing day (last day), one of the two identical objects used during the training phase (familiar object) was replaced by a new different object (the novel object) and located in the same spot as the one before. Object exploration was scored when the mouse’s nose was within 2 cm of the object and the vibrissae were moving towards it [54]. The experiment was recorded using a webcam placed 1.5 m above the arena. The exploratory behavior was manually scored in blind by home-made software Behavior Scoring Panel® 2008 by A. Dubreucq Version 3.0 beta. Preference for the novel object was calculated by the discrimination index, using the formula [(time devoted to the novel object – time devoted to the familiar object) / (time devoted to the novel object + time devoted to the familiar object)]. This is the parameter for the evaluation of long-term object recognition memory that was used as a proxy of cognitive impairment.

### General Stereotaxic Surgical Procedures

Mice were anesthetized with isoflurane gas using a CA-ECA20 Anesthesia Trolley System equipment (Cibertec, Madrid, Spain), and placed for stereotaxic surgery using a Kopf™ stereotaxic frame (David Kopf Instruments, Tujunga, CA, USA) with the head horizontally immobilized. An incision into the scalp was made to reveal lambda and bregma suture points. One microdialysis probe (2.0 × 0.25 mm) was implanted in the ipsilateral PFC (anterior-posterior (A/P): +2.0 mm; medial-lateral (M/L): +0.3 mm; dorso-ventral (D/V): -3.3 mm, coordinates taken from bregma). These coordinates were chosen using the Paxinos and Franklin atlas [55]. After that, probes were fixed to the skull with dental cement and protected with a plastic cover to prevent probe damage. Once recovered from surgery, mice were housed individually with water and food *ad libitum*.

### Microdialysis Procedures and Drug Administration

All the microdialysis experiments were performed between 20 and 24 h after probe implantation. Sampling was carried out using Raturn Microdialysis System (BASi, West Lafayette, IN, USA), allowing animals to freely move in the cage. After the recovery period, probes were connected to the system and artificial cerebrospinal fluid (aCSF) solution was perfused through the probes at a rate of 1 µl/min incessantly during the whole experiment. The composition of the aCSF was 148 mM NaCl, 2.7 mM KCl, 1.2 mM CaCl_2_ and 0.85 mM MgCl_2_ with pH of 7.4, adjusted with 1 mM K_2_HPO_4_. Following 1 h of stabilization, aCSF was perfused through the probes and dialysate samples were collected every 35 min in refrigerated vials containing 5 µl acetic acid (0.1 M) [56, 57]. Six samples were obtained before drug administration.

Afterward, amphetamine (2.5 mg/kg) or dizocilpine (MK-801) (0.5 mg/kg) were systemically administered (dissolved in saline solution at 5 ml/kg i.p.). Control animals received saline (5 ml/kg i.p.). In addition, aCSF enriched in K^+^ (100 mM NaCl, 50 mM KCl, 1.2 mM CaCl_2_ and 0.85 mM MgCl_2_ with a pH of 7.4 adjusted with 1 mM K_2_HPO_4_) was also administered locally in the PFC. Following drug administration, a number between seven and eleven additional samples was collected.

#### Non-Net Flux Microdialysis

In the case of the non-net flow microdialysis assays, the composition of the aCSF also included 0.25 mM ascorbic acid as an antioxidant, to avoid catecholamine degradation during the experiment. For these assays, the pH was adjusted between 7.0 and 7.2 with NaOH 0.5 N. After 1 h of stabilization, samples were collected every 35 min in vials containing 5 µl perchloric acid (0.1 M). The experiment started collecting between 1 and 3 samples that were not used neither in the analysis nor in the subsequent interpretation of the results, but this procedure allowed to guarantee the stabilization of the neurotransmitter concentrations in the specific brain area. Then, different solutions of the neurotransmitter of interest (NA or DA) were randomly perfused through the probe at concentrations of 0, 5, 10 and 20 nM (C_*in*_). When changing the concentration solutions, a 70-min waiting time was left before starting to collect the next sample, since it is considered the time necessary to reach a steady or equilibrium state. Once this state was reached, two samples were collected for each of the administered concentrations and three samples after the final administration. These samples were used to evaluate the neurotransmitter concentrations in the dialysate (C_*out*_). After the experiments, mice were sacrificed by cervical dislocation.

### Brain Tissue Catecholamine Analysis

Mice were sacrificed by cervical dislocation, brains quickly removed, and the whole cortex was dissected and weighted. Immediately, samples were placed in glass tubes and homogenized in cold buffer containing 0.1 N perchloric acid with 100 µM EDTA (15 µl/mg of tissue) using a T-10 basic Ultra-Turrax® (IKA, Staufen, Germany). Then, the tubes were centrifuged at 20,817 RCF/ *g*-force for 15 min at 4 °C. Subsequently, the supernatants were filtered using Costar Spin-X™ Centrifuge filters 0.22 μm (Sigma-Aldrich, St. Louis, MO, USA) at 1000 RCF/ *g*-force during 5 min at 4 °C. From the obtained final filtrates, 40 µl of each sample was separated for the immediate catecholamine analysis by HPLC and results were expressed as nM/mg tissue. All the samples were processed freshly on the same day.

### Chromatographic Analysis and Determination of Catecholamine Concentration

For microdialysis assays, catecholamine concentration in the samples was determined using ultra-high-performance liquid chromatography (UHPLC) with amperometric detection (SENCELL™, Decade Elite, Antec Scientific, Zoeterwoude, The Netherlands) at an oxidizing potential of 0.46 V. The mobile phase was composed of 100 mM phosphoric acid, 100 mM citric acid, 0.1 mM EDTA, 950–1500 mg/l 1-octane sulfonic acid (OSA), 5% v/v acetonitrile, and Milli-Q® water. The pH was adjusted up to 6.0 with a 50% NaOH/45% KOH solution. The mobile phase was degassed for 10–15 min in a sonic bath and flowed through the system at a rate of 0.075 ml/min in an Acquity UPLC BEH C18, 1.7 μm, 1 × 100 mm (Waters, Milford, MA, USA) chromatographic column. Samples with a final volume of 40 µl (35 µl dialysate + 5 µl acetic acid (0.1 M)) were placed in the AS 110 autosampler. The injection volume in the UHPLC equipment was 13 µl. Catecholamine peak areas were simultaneously detected at the same sample running time and measured using the Clarity software (DataApex, Prague, Czech Republic). The detection limit of the assay was 0.05 nM approximately per sample.

For tissue content and non-net flow microdialysis assays, catecholamine concentration in the samples was determined using HPLC equipment with amperometric detection (VT-03 cell, Decade II, Antec Scientific, Zoeterwoude, The Netherlands) at an oxidizing potential of 0.30 V. The separation was carried out with a mobile phase composed of 50 mM phosphoric acid, 0.1 mM EDTA, 8 mM NaCl, 500 mg/l sodium octyl sulfate, and 5–18% methanol. The pH 6.0 was adjusted with NaOH 6 N. The mobile phase was filtered and delivered at a flow rate of 0.2 ml/min in an ALF-215, 2.1 × 150 mm, C18 (Antec Scientific, Zoeterwoude, The Netherlands) chromatographic column by a model 1100 pump (Agilent Technologies, Madrid, Spain) after degasification (Agilent Technologies model 1100 degasser). A 40 µl (35 µl dialysate + 5 µl 0.1 M perchloric acid) (microdialysis) or 40 µl (tissue content assay) volume of each sample was collected in each microvial and an injection volume of 30 µl was injected in the HPLC system. The peak areas of NA and DA were integrated by a Chemstation plus Software (Hewlett Packard Ltd., Morain, OH, USA). The detection limit of the assay was approximately 0.1 nM per sample.

### Protein Expression by Western Blot

#### Sample Preparation

Frontal cortex samples of saline and poly(I:C) mice were processed as total homogenates for Western blot experiments. Hundred milligrams of brain tissue were homogenized in 1 ml of cold Tris-sucrose buffer (0.32 M sucrose in 5 mM Tris-HCl, pH 7.4) supplemented with protease and phosphatase inhibitors (50 µL/g of Sigma protease inhibitor Cocktail, 5 mM Na_3_VO_4_, and 10 mM NaF) using a mechanical tissue homogenizer at 4 °C. Protein content was measured using the Bradford method. The samples of saline and poly(I:C) mice belonging to the same litter were processed on the same day.

#### Western Blot Technique

Samples were prepared in electrophoresis buffer in reducing and denaturing conditions (100 mM DTT, 2% SDS, 8% glycerol, 0.01% bromophenol blue and heated at 95 °C for 5 min). Denatured proteins (10–20 µg) were resolved on 12% SDS-PAGE gels and transferred to nitrocellulose membranes. After being blocked for 1 h at room temperature (5% non-fat dry milk in PBS), membranes were incubated overnight at 4 °C with constant agitation with the primary antibodies against dopamine transporter (DAT) (Merk Millipore MAB369, 1:500, Darmstadt, Germany), noradrenaline transporter (NET) (MabTechnologies NET05-2, 1:1000, Neenah, WI, USA), tyrosine hydroxylase (TH) (Santa Cruz SC25269, 1:500, Dallas, TX, USA), dopamine D_1_ receptor (D_1_R) (Abcam AB78021, 1:500, Cambridge, MA, USA), dopamine D_2_ receptor (D_2_R) (Santa Cruz SC5303, 1:500, Dallas, TX, USA) or β-actin (Sigma-Aldrich A1978, 1:200,000, Sigma-Aldrich, St. Louis, MO, USA or Abcam AB8227, 1:10,000, Cambridge, MA, USA). After incubation with the fluorescent secondary antibodies (IRDyeTM 800 or Alexa Fluor® 680 conjugated) for 1 h at room temperature, the immunoreactive signal (integrated intensity values) was detected using the Odyssey infrared imaging system (LI-COR Biosciences) and quantified using Image Studio Lite 5.2 (LI-COR Biosciences). A standard pool of total homogenate was processed in the same gels and used as external reference sample. The immunoreactivity values were normalized for the β-actin signal. Antibody selection criteria was included in Additional file 2. Whole uncropped images of the original Western blots from which figures have been derived is shown in Additional file 3: Supplementary Fig. 1).

### Statistical Analysis

All experiments were randomized and analyzed in blind. The required sample sizes were estimated on the basis of our past experience performing similar experiments and power analysis calculations performed with GPower 3.1.9.7 software (University of Düsseldorf, Germany).

For behavioral assays, catecholamine tissue concentration analysis and Western blot experiments, data are expressed in bars as mean ± the standard error of the mean (SEM). Student’s unpaired *t*-test was used for the comparison between saline and poly(I:C) mice. In addition, repeated two-way ANOVA was used to evaluate the familiar and novel object exploration time between saline and poly(I:C) mice. The level of significance was taken as *p* < 0.05.

In microdialysis experiments, the last two from the first six collected samples were taken into account to establish the mean basal concentration values (baseline), and were considered the 100%. The effect triggered by the administration of the drug of interest was calculated and represented as percentage of this baseline. The maximal effect (Emax) over basal values achieved by each drug administration is shown. Results are expressed as the mean values ± SEM values of n individual animals. Basal values between groups were compared by an unpaired Student’s *t*-test. One-way analysis of variance (ANOVA) followed by Dunnett’s *post hoc* test was used to assess the effect of each drug over time. Two-way repeated-measures ANOVA, with treatment (saline vs. poly(I:C)) groups as the independent variables and time as the repeated-measure factor, was used to evaluate whether an interaction between these two variables on the dependent variable existed. It was followed by a Bonferroni’s *post hoc* test for individual sample comparison. F values are expressed as F_treatment_ (F_tr_), which expresses the ability of the treatment to exert an effect, F_time_ (F_t_) that indicates effects across time and, F_interaction_ (F_i_), which shows the differences between the two groups of treatment across time. For these analyses, basal values and the samples after the administration of the drug were taken into consideration. Results were represented as mean values ± SEM. The minimum statistical significance was set at *p* < 0.05.

In non-net flow microdialysis experiments [58], the difference between the neurotransmitter concentration applied through the probe (C_*in*_) and the concentration of neurotransmitter present in the dialysate after the determined concentration perfusion (C_*in*_-C_*out*_) is represented. C_*out*_ was obtained by calculating the mean value of the neurotransmitter concentrations present in the two samples collected for each administered standard pattern. Through a linear regression of the obtained data, the extracellular concentrations of the neurotransmitter and the extraction fraction (Ed) were calculated individually for each animal. The value of C_*in*_ to which C_*in*_-C_*out*_=0 corresponds to the conditions of the equilibrium state and represents the concentration of the neurotransmitter in the extracellular fluid (*non-net flow point*). Also, using this linear regression, the value of the slope of the line or Ed was calculated, considered a direct function of the clearance of the neurotransmitter from the extracellular space. Data are expressed as the mean values of each group ± SEM. The analysis of the comparison of both parameters in the two groups was carried out by a two-tailed unpaired Student’s *t*-test. All the graphs and statistical analyses were carried out using GraphPad Prism™ software (GraphPad Software, San Diego, CA, USA).

## Results

### Cognitive Ability of Saline and Poly(I:C) Mice in the Novel Object Recognition Test

**Fig. 1:**
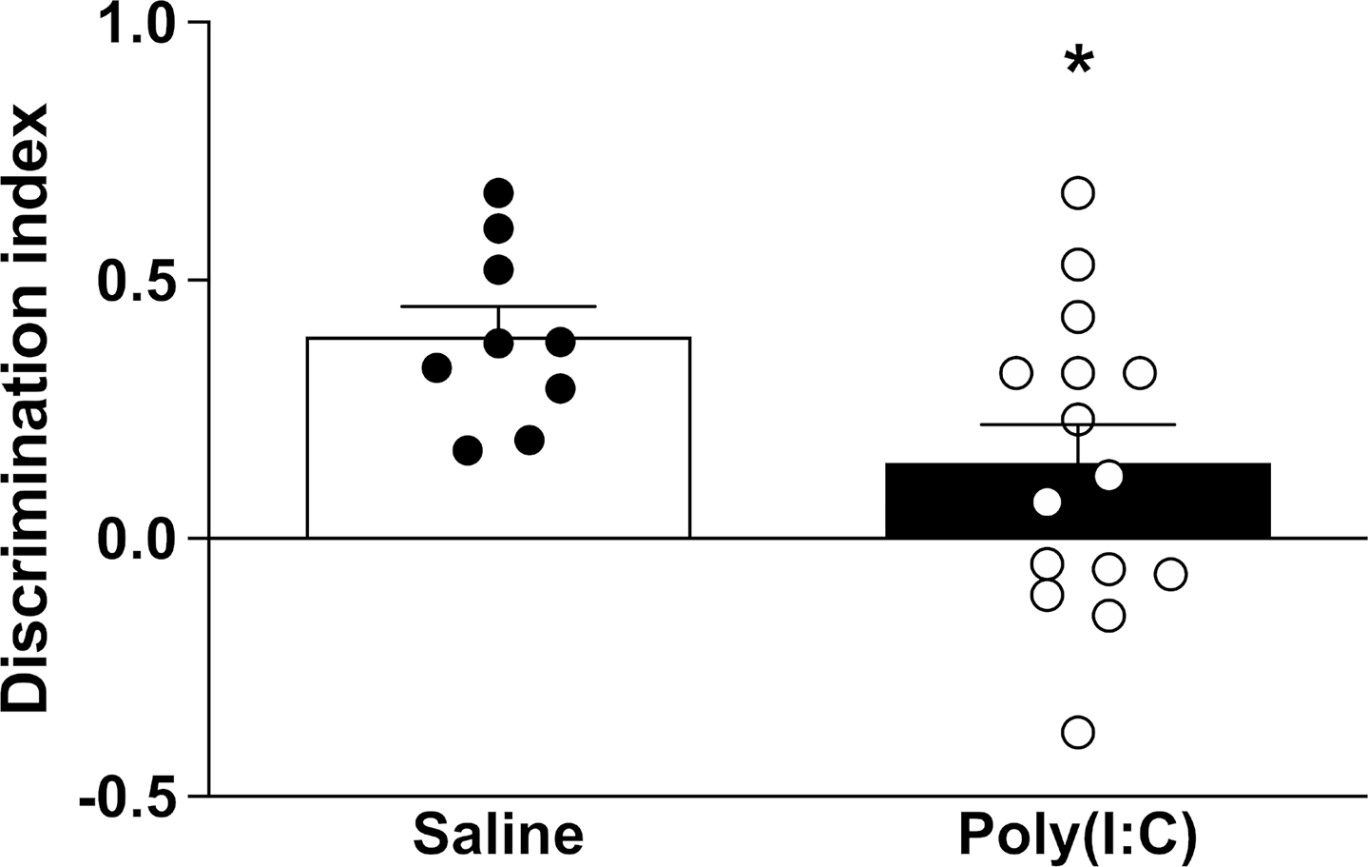
Discrimination index as indicative parameter of cognitive status. Representation of the discrimination index of saline control (n = 9) and poly(I:C) (n = 15) mice evaluated by NORT. Bars represent mean ± SEM values. *p < 0.05, Student’s unpaired *t*-test vs. control group

Offspring of poly(I:C) mice presented a lower discrimination index (*t* = 2.30, *p* = 0.031) compared to the saline mice (Fig. 1). Individual parameters to calculate discrimination index are shown in Additional file 4: Supplementary Fig. 2. On the test day, both saline and poly(I:C) mice spent similar time exploring the familiar object, with no differences between groups (*t* = 0.305, *p* = 0.763) (Additional file 4: Supplementary Fig. 2A). As for the novel object, a lower exploration time was observed in the poly(I:C) compared to the saline group (*t* = 1.73, *p* = 0.096), although it did not reach statistical significance (Additional file 4: Supplementary Fig. 2B). Thus, the total time that both groups of mice spent exploring both the familiar and the novel objects was not different (*t* = 1.32, *p* = 0.198), even if poly(I:C) offspring exhibited a tendency to a shorter exploration time than saline mice (Additional file 4: Supplementary Fig. 2C). Comparison of familiar and novel exploration times between saline and poly(I:C) animal model performed by two-way ANOVA followed by Bonferroni *post* *hoc* test showed differences in the discrimination of objects in the saline group (p = 0.0004) but not in poly(I:C) MIA model (Additional file 5: Supplementary Fig. 3).

### Characterization of Basal Extracellular Catecholamine Concentrations and Extraction Fraction (Ed) in the PFC of Awake Saline and Poly(I:C) Mice by Non-Net Flow Microdialysis

**Fig. 2:**
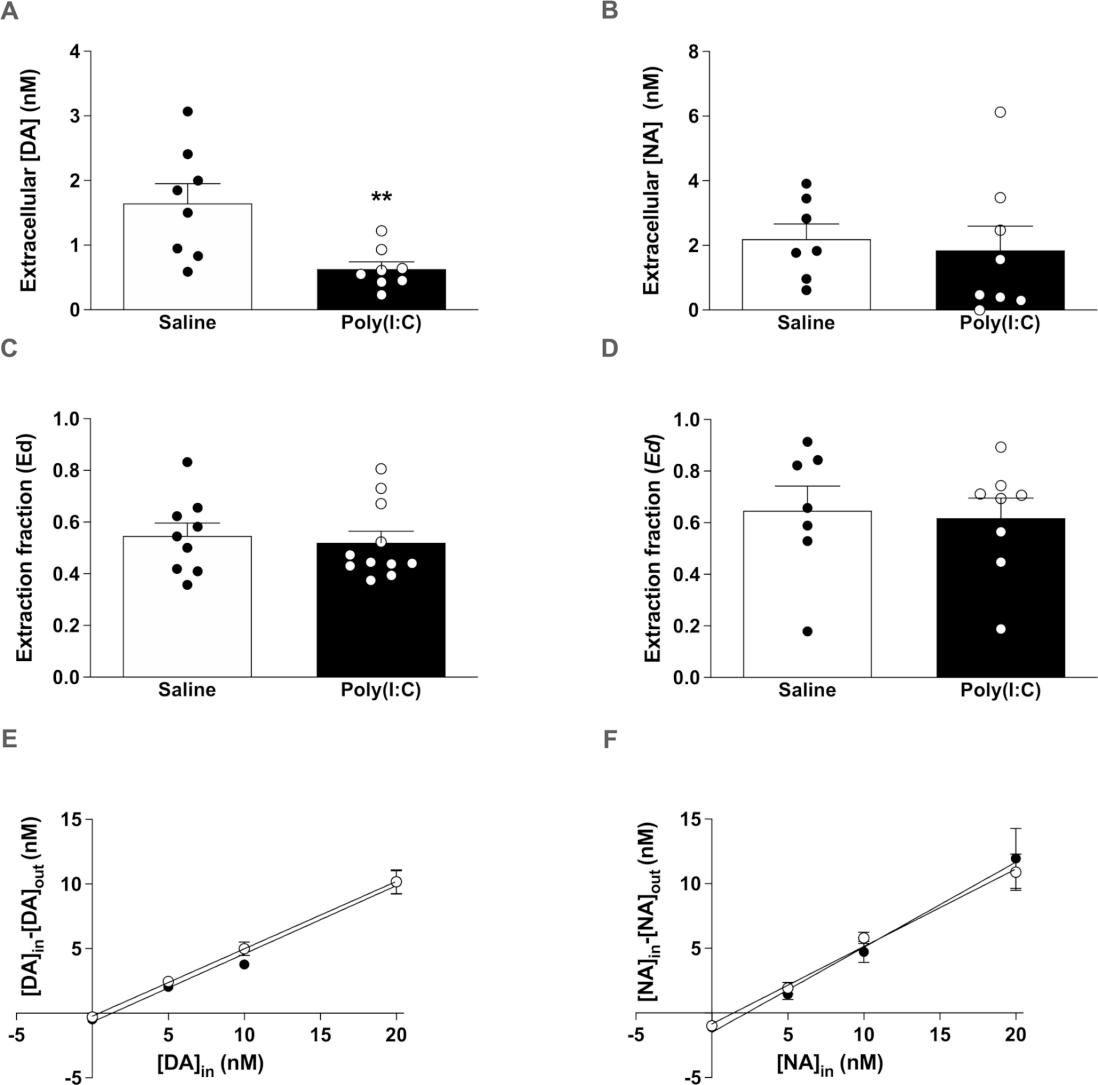
Non-net flux microdialysis. Representations of: **(A)** extracellular DA concentrations in PFC of saline control (n = 8) and poly(I:C) (n = 8) mice as indicated by the point of non-net flow microdialysis; **(B)** extracellular NA concentrations in PFC of saline control (n = 7) and poly(I:C) (n = 8) mice as indicated by the point of non-net flow microdialysis; **(C) **in vivo DA extraction fraction (Ed) in PFC of saline control (n = 9) and poly(I:C) (n = 11) mice; **(D) **in vivo NA extraction fraction (Ed) in PFC of saline control (n = 7) and poly(I:C) (n = 8) mice; **(E)** Non-net flow cerebral microdialysis assay for DA in PFC of saline control (n = 9) (●) and poly(I:C) (n = 11) (○) mice; **(F)** Non-net flow cerebral microdialysis assay for NA in PFC of saline control (n = 7) (●) and poly(I:C) (n = 8) (○) mice. Bars represent mean ± SEM values. In the linear regressions (**E** and **F**), the value of C_*in*_ to which C_*in*_-C_*out*_=0 represents the concentration of the neurotransmitter in the extracellular fluid (*non-net flow point*) and the value of the slope indicates Ed. **p < 0.01, Student’s unpaired *t*-test vs. saline group

There was a statistically significant decrease in the basal extracellular concentrations of DA showed by poly(I:C) mice (0.63 ± 0.11 nM) compared to the control group (1.65 ± 0.30 nM) (*t* = 3.17, *p* = 0.0068) (Fig. 2A and E). However, regarding the Ed, poly(I:C) mice presented a mean value of 0.52 ± 0.04 and the saline group 0.55 ± 0.05. There were no statistically significant differences between groups (*t* = 0.24, *p* = 0.8134) (Fig. 2C and E). The Ed value represents the reuptake function that the neurotransmitter undergoes by its transporters (DAT and NET), but it is also indicative of processes such as diffusion, metabolic activity of enzymes including catechol-o-methyltransferase (COMT), and uptake by low-affinity transport mechanisms such as organic cation transporters (OCTs) and plasma membrane monoamine transporters (PMATs) [59].

There were no statistically significant differences between the basal NA values presented by poly(I:C) mice (1.85 ± 0.75 nM) and the respective saline group (2.20 ± 0.47 nM) (*t* = 0.38, *p* = 0.7102) (Fig. 2B F). Regarding the Ed, poly(I:C) mice presented a mean value of 0.62 ± 0.08 and the saline group 0.65 ± 0.09, showing no significant differences between groups (*t* = 0.24, *p* = 0.8134) (Fig. 2D F).

### K^+^-Evoked Release of Catecholamines in the PFC by the Local Administration of HyperK^+^ aCSF

**Fig. 3:**
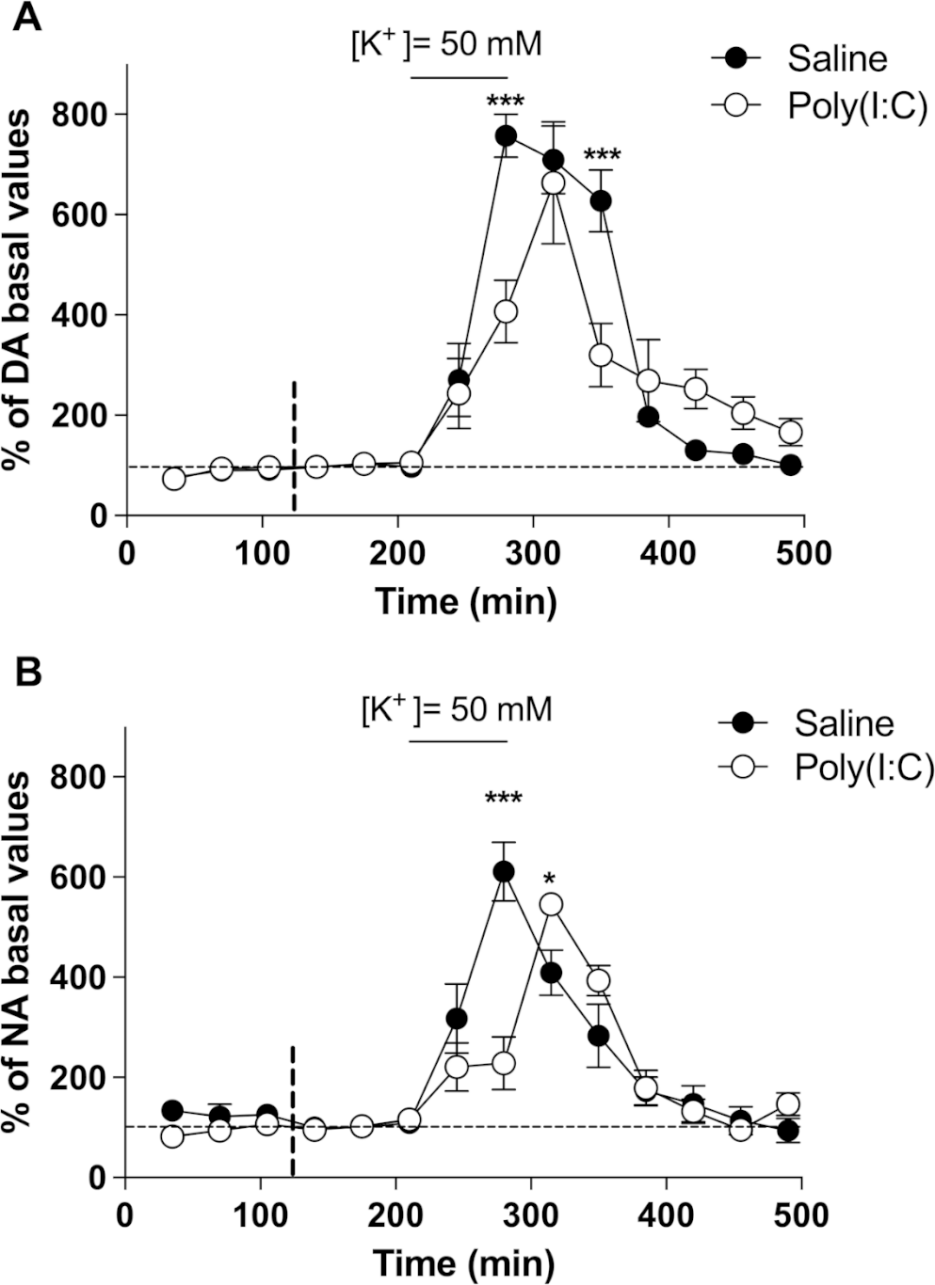
Potential-dependent catecholamine release. Representation of the effect of local administration in PFC of hyperK^+^ aCSF (50 mM) during 105 min on extracellular concentrations of **(A)** DA and **(B)** NA in PFC of saline control (●) (n = 6) and poly(I:C) (○) mice (n = 5). The upper horizontal continuous line indicates the time interval of administration of hyperK^+^ aCSF. The vertical discontinuous line marks the point from which the basal concentrations have been taken into consideration. Points represent mean ± SEM values and are expressed as percentages of basal values. *p < 0.05, ***p < 0.001, two-way ANOVA followed by Bonferroni *post hoc* test

Local administration of hyperK^+^ aCSF forces the global depolarization of neuronal membranes and eventually induces the release of neurotransmitters. HyperK^+^ aCSF (50 mM) into the PFC evoked a significant increase in DA extracellular concentration in saline mice (Emax = 757 ± 43%; *p* < 0.001 vs. basal conditions) (Fig. 3A). This maximum effect occurred 70 min after the beginning of the hyperK^+^ perfusion (F[10,50] = 49.39, p < 0.0001, n = 6). For poly(I:C) group, local PFC administration of hyperK^+^ aCSF also increased DA concentrations significantly (Emax = 663 ± 121%; *p* < 0.001 vs. basal conditions), and this maximum effect was reached 105 min after the beginning of the perfusion (F[10,40] = 9.952, *p* < 0.0001, n = 5). Two-way ANOVA for repeated measures revealed significant differences between groups (F_t_[10,90] = 43.33, *p* < 0.0001; F_tr_[1,90] = 1.224, *p* = 0.2972; F_i_[10,90] = 5.916, *p* < 0.0001; n = 11).

Similarly, the local PFC administration of hyperK^+^ aCSF (50 mM) induced a significant increase in NA concentration in saline mice (Emax = 611 ± 59%; *p* < 0.001 vs. basal conditions). This maximum effect was reached 70 min after the beginning of hyperK^+^ perfusion (F[10,50] = 22.64, *p* < 0.0001, n = 6) (Fig. 3B). In poly(I:C) mice group, the local administration in PFC of hyperK^+^ aCSF also increased NA concentrations (Emax = 546 ± 17%; *p* < 0.001 vs. basal conditions) and the maximum effect occurred 105 min after the beginning of the perfusion (F[10,40] = 25.80, *p* < 0.0001, n = 5). Two-way ANOVA for repeated measures revealed significant differences between groups (F_t_[10,90] = 36.27, *p* < 0.0001; F_tr_[1,90] = 1.841, *p* = 0.208; F_i_[10,90] = 8.686, *p* < 0.0001; n = 11).

### Effect of the Systemic Administration of Amphetamine on Catecholamine Concentration in the PFC

**Fig. 4:**
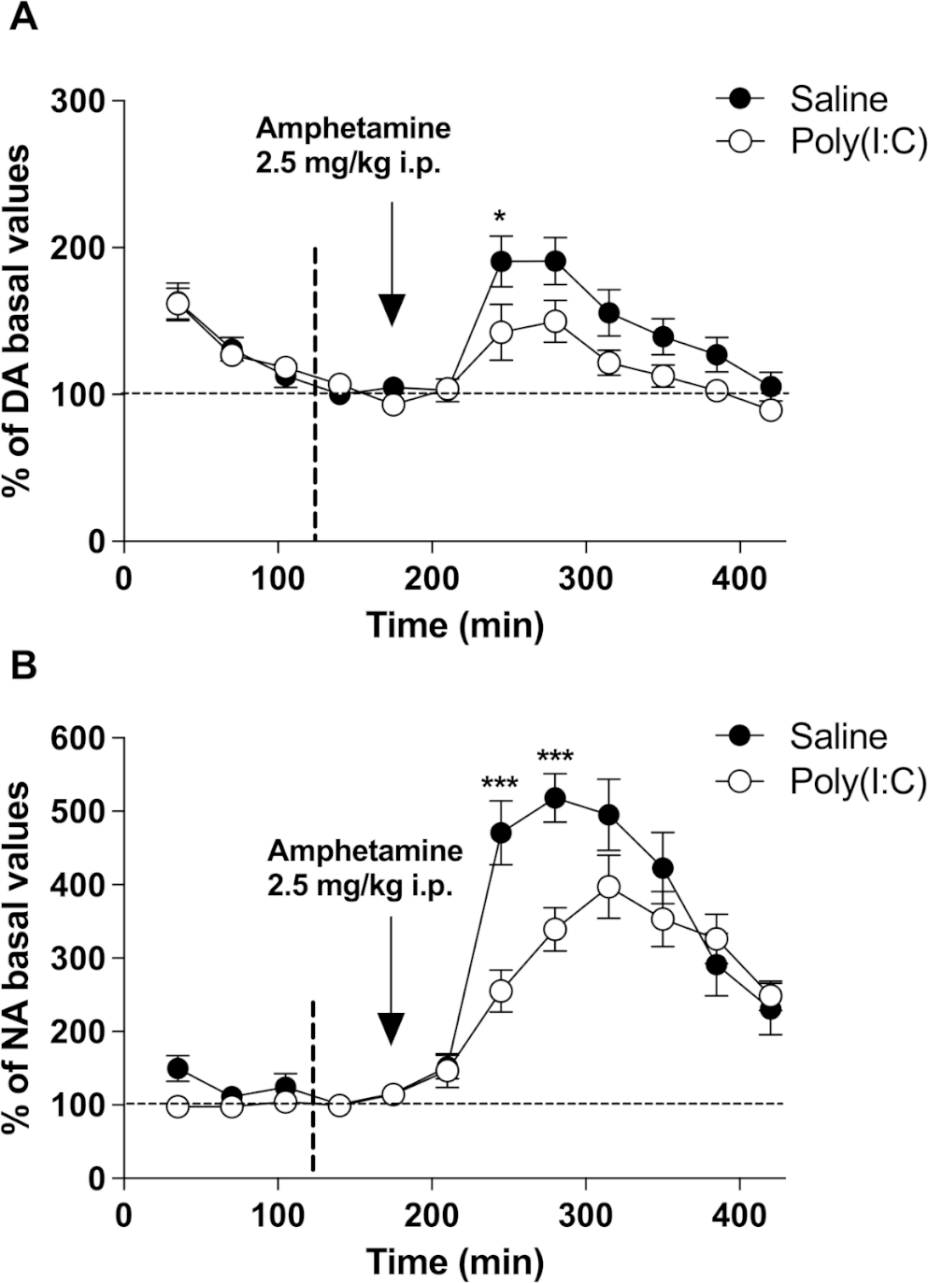
Potential-independent catecholamine release. Representation of the effect of systemic administration of amphetamine (2.5 mg/kg i.p.) on extracellular concentrations of **(A)** DA and **(B)** NA in PFC of saline control (●) (n = 22) and poly(I:C) (○) mice (n = 21). The vertical arrow indicates the time of administration of amphetamine. The vertical discontinuous line marks the point from which the basal concentrations have been taken into consideration. Points represent mean ± SEM values and are expressed as percentages of basal values. *p < 0.05, ***p < 0.001, two-way ANOVA followed by Bonferroni *post hoc* test

Systemic administration of amphetamine (2.5 mg/kg i.p.) evoked a significant increase of extracellular DA concentration in the PFC of saline mice (Emax = 191 ± 16%; *p* < 0.001 vs. basal conditions) (Fig. 4A). This maximum effect occurred 70 min after injecting the drug (F[8,168] = 15.84, *p* < 0.0001, n = 22). In the poly(I:C) group, the systemic administration of amphetamine also increased DA concentrations significantly (F[8,160] = 7.237, *p* < 0.0001, n = 21), reaching a maximum effect of 150 ± 14% (*p* < 0.001 vs. basal conditions) 105 min after the injection. Two-way ANOVA for repeated measures showed significant differences between groups (F_t_[8,328] = 22.01, *p* < 0.0001; F_tr_[1,328] = 4.507, *p* = 0.040; F_i_[8,328] = 2.319, *p* = 0.020; n = 43).

Regarding NA, systemic administration of amphetamine (2.5 mg/kg i.p.) induced a significant increase of NA concentration in PFC of saline mice (Emax = 518 ± 33%; *p* < 0.001 vs. basal conditions). The maximum effect was reached 105 min after the injection of the drug (F[8,160] = 32.49, *p* < 0.0001, n = 21) (Fig. 4B). In the PFC of poly(I:C) mice, the systemic administration of amphetamine also increased NA concentrations significantly (F[8,168] = 22.34, *p* < 0.0001, n = 22), with a maximum effect of 397 ± 43% (*p* < 0.001 vs. basal conditions) reached 140 min after the injection. Two-way ANOVA for repeated measures revealed statistically significant differences between both groups (F_t_[8,328] = 52.07; *p* < 0.0001; F_tr_[1,328] = 4.322; *p* = 0.044; F_i_[8,398] = 5.727; *p* < 0.0001; n = 43).

### Effect of the Systemic Administration of the NMDA Receptor Antagonist MK-801 on Catecholamine Concentrations in the PFC

**Fig. 5:**
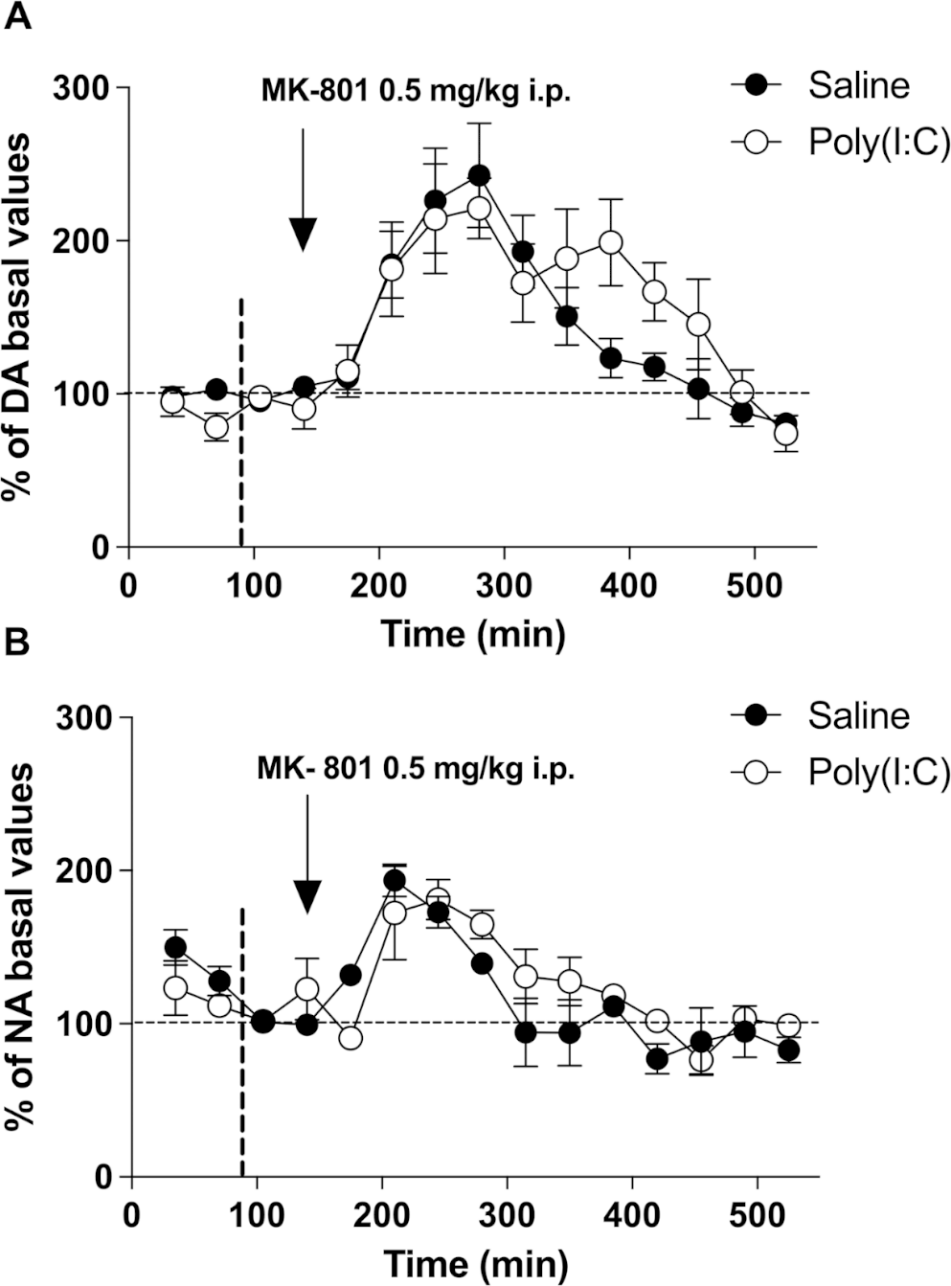
MK-801 induced chatecholamine release. Representation of the effect of systemic administration of MK-801 (0.5 mg/kg i.p.) on extracellular concentration of **(A)** DA and **(B)** NA in PFC of saline control (●) (n = 5–6) and poly(I:C) (○) mice (n = 5). The vertical arrow indicates the time of administration of MK-801. The vertical discontinuous line marks the point from which the basal concentrations have been taken into consideration. Points represent mean ± SEM values and are expressed as percentages of basal values

In order to identify whether the excitatory drive of glutamatergic pyramidal cells on catecholaminergic pathways could be involved in the reduced DA and NA cortical release of MIA offspring, the in vivo response to administration of the NMDA receptor antagonist MK-801 was assessed. In animals, administration of different NMDA receptor antagonists such as phencyclidine (PCP), ketamine or MK-801 leads to cognitive deficits similar to those observed in schizophrenia [60]. The systemic administration of the NMDA receptor antagonist MK-801 (0.5 mg/kg i.p.) resulted in a significant increase in DA concentrations in the PFC of saline offspring (Emax = 243 ± 34%; *p* < 0.001 vs. basal conditions) (Fig. 5A), reaching maximum effect 140 min after the injection (F[12,60] = 11.05, *p* < 0.0001, n = 6). In poly(I:C) mice, DA concentration was also significantly augmented by MK-801, with a maximum effect of 221 ± 20% (*p* < 0.01 vs. basal conditions) (F[12,48] = 5.881, *p* < 0.0001, *n* = 5) obtained 140 min after the injection of this drug. Two-way ANOVA for repeated measures showed no significant differences between groups (F_t_[12,108] = 14.81; *p* < 0.0001; F_tr_[1,108] = 0.498; *p* = 0.498; F_i_[12,108] = 1.368; *p* = 0.192; n = 11).

In the case of NA, systemic administration of MK-801 (0.5 mg/kg i.p.) induced a significant increment of NA concentration in the PFC of saline mice (Emax = 194 ± 10%; *p* < 0.001 vs. basal conditions) (Fig. 5B). The highest effect on NA concentrations was reached 70 min after the injection (F[12,48] = 7.758, *p* < 0.0001, n = 5). In poly(I:C) mice, the NMDA receptor antagonist was also capable of increasing NA concentration significantly in the PFC, producing a maximum effect of 181 ± 13% (*p* < 0.05 vs. basal conditions) 105 min after the administration of the drug (F[12,48] = 1.133, *p* < 0.0001, n = 5). Two-way ANOVA for repeated measures did not reveal significant differences between groups (F_t_[12,96] = 12.21; *p* < 0.0001; F_tr_[1,96] = 2.196; *p* = 0.1767; F_i_[12,96] = 1.485; *p* = 0.1431; n = 10).

### Catecholamine Tissue Concentration in the Brain Cortex of Saline and Poly(I:C) Mice

DA tissue content in the whole brain cortex were 535.4 ± 55.12 nM/mg tissue (n = 21) in saline mice and 474.7 ± 47.91 nM/mg tissue in poly(I:C) mice (n = 19). NA brain cortex contents were 64.27 ± 5.47 nM/mg tissue in saline mice (*n* = 21) and 59.94 ± 5.95 nM/mg tissue in poly(I:C) mice (*n* = 20). There were no statistical differences in DA or NA tissue concentrations between both groups of animals (*t* = 0.823, *p* = 0.416; *t* = 0.537, *p* = 0.594, respectively) (Additional file 6: Supplementary Fig. 4).

### Protein Expression in the Brain Frontal Cortex of Saline and Poly(I:C) Mice

In order to assess whether poly(I:C) mice showed molecular alterations in brain catecholaminergic systems, the protein expression of DAT, NET, TH, D_1_R and D_2_R was evaluated by Western blot in the frontal cortex of saline (*n* = 23) and poly(I:C) offspring mice (*n* = 27).

**Fig. 6:**
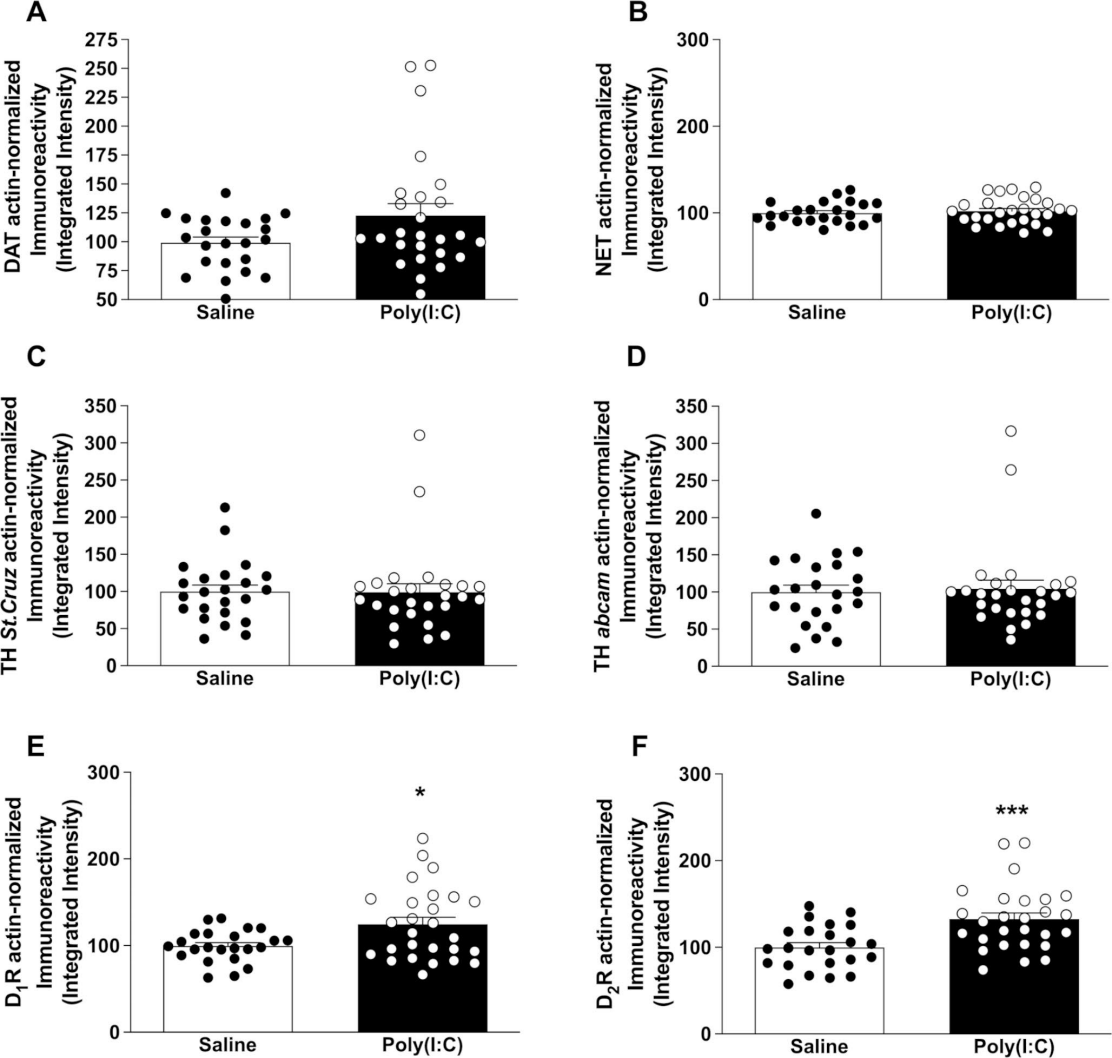
Western blot of different targets of catecholamine neurotransmission systems. Representation of immunodetection values in brain cortex of saline control (n = 23) and poly(I:C) (n = 26–27) mice for **(A)** DAT expression; **(B)** NET expression; **(C)** TH expression by Santa Cruz antibody; **(D)** TH expression by Abcam antibody; **(E)** dopamine D_1_R expression; and **(F)** dopamine D_2_R expression. Bars represent mean ± SEM values of independent experiments and express the β-actin-normalized immunoreactivity levels as percentage of the external reference sample. *p < 0.05, ***p < 0.001, Student’s unpaired *t*-test

The quantification of DAT immunoreactivity demonstrated no significant differences between poly(I:C) (123 ± 10%) and saline (100 ± 5%) mice (*t* = 1.99, *p* = 0.052), although a trend to increased expression was observed in poly(I:C) group (Fig. 6A). Similarly, NET protein expression showed no significant differences between poly(I:C) (102 ± 3%) and saline (100 ± 3%) mice (*t* = 0.55, *p* = 0.586) (Fig. 6B). Regarding TH, the limiting enzyme in the catecholamine synthesis, very similar results were obtained with the two antibodies. Thus, no significant differences were detected in the TH immunoreactivity with the Santa Cruz antibody between poly(I:C) (99 ± 11%) and saline (100 ± 9%) groups (*t* = 0.06, *p* = 0.955) (Fig. 6C). In the same way, TH levels measured with the Abcam antibody revealed no statistical differences between poly(I:C) (104 ± 12%) and saline (100 ± 9%) mice (*t* = 0.27, *p* = 0.783) (Fig. 6D).

The quantification of the D_1_R immunoreactivity demonstrated a significant increase in poly(I:C) mice compared to the saline group (*t* = 2.64, *p* = 0.011) (Fig. 6E). D_1_R expression levels were 100 ± 4% for saline and 125 ± 8% for poly(I:C) groups. Likewise, D_2_R protein expression also presented a statistically significant increase in poly(I:C) mice compared to saline mice (*t* = 3.55, *p* = 0.0009) (Fig. 6F). D_2_R expression levels were 100 ± 5% for the saline and 133 ± 7% for the poly(I:C) group.

Lastly, potential correlations between different immunoreactivities were evaluated. Regarding the TH, a significant positive correlation between the Santa Cruz and the Abcam TH antibodies was observed (*r* = 0.92, *n* = 49, *p* < 0.0001) (Additional file 7: Supplementary Fig. 5A), granting robustness to the results obtained with the two different antibodies. In addition, when the immunoreactivity signal relationship for TH and DAT was analyzed, a positive correlation was detected for both TH Santa Cruz (r = 0.63, n = 49, p < 0.0001) and TH Abcam antibodies (r = 0.68, n = 49, p < 0.0001) (Additional file 7: Supplementary Fig. 5B and 5 C). These results suggest a correlation between the proteins responsible for the DA synthesis and turnover. Finally, the D_2_R and D_1_R antibodies also showed a positive correlation between them (*r* = 0.74, *n* = 50, *p* < 0.0001) (Additional file 7: Supplementary Fig. 5D), indicating a similar pattern of alteration in the expression of both receptors in the brain frontal cortex.

## Discussion

The present results demonstrate that prenatal immune challenge impairs the correct functioning of dopaminergic and noradrenergic pathways in the PFC, and affects cognitive behavior in offspring, probably by shifting the normal neurodevelopmental trajectory. Specifically, these findings provide evidence that MIA during early prenatal development induces hypofunction of presynaptic DA- and NA-evoked release in the brain cortex, leading to reduced synaptic concentrations of DA in the area. Because similar alterations in DA neurotransmission have been reported and associated to negative and cognitive dysfunctions in schizophrenia, the poly(I:C) MIA model reported here could be considered as a valuable translational tool for the study of this disorder. Consistent with the involvement of cortical catecholamine alterations in cognitive impairment, this mouse model displayed deficits in the visuospatial learning and/or the memory evocation capacity when performing object recognition tasks.

The marked variations reported in poly(I:C)-induced MIA protocols represent an important source of the experimental variability [61]. Among others, differences in gestational age at immunization time, type of immunogen, dose of poly(I:C), route of delivery, sex, animal species and strains represent conditions that confer heterogeneous susceptibility to MIA in the offspring [34, 47, 62–66]. However, even if the poly(I:C) protocol may differ across studies, cognitive deficits in offspring seem to be a consistent and relevant feature of this MIA model. Thus, the existence of an aberrant cognitive behavior helps to confirm the previous transmission of harmful factors from exposed mothers to their offspring, resulting in perturbed neurodevelopment. In addition to altered behavioral patterns, the achievement of a maternal immune response elicited under particular experimental conditions of the present study was also confirmed by the reduction of maternal body temperature and loss of maternal body weight gain [67].

The NORT is a reliable test used previously by several groups to measure cognitive deficits in different MIA animal models. NORT evaluates episodic-like declarative memory, one of the cognitive domains considered by the MATRICS initiative to be deficient in schizophrenia [68]. Across numerous studies, the poly(I:C) model has exhibited disrupted recognition memory and lower discrimination index both in mice [52, 69–72] and rats [73–76]. Other cognitive domains relevant to schizophrenia have also been described to be altered in offspring of poly(I:C)-based MIA models [33, 34, 77]. Therefore, neurodevelopment disruptions induced by poly(I:C) seem to entail behavioral impairments in rodents that satisfy face validity criteria for schizophrenia-like symptoms.

Neuroimaging studies that evaluate binding potential of D_2_R radiotracers have shown that administration of catecholamine-releasing stimulants as amphetamine promotes an excessive release of DA in the striatum of schizophrenia subjects [78–81]. In contrast, in PFC and other cortical brain areas, the effect of catecholamine-releasing drugs has demonstrated a blunted DA release in schizophrenia [15, 16]. These observations point to the existence of cortical hypodopaminergia in schizophrenia that could have its origin at presynaptic level [79]. Association between DA release capacity and working memory activation has been detected in the dorsolateral PFC, suggesting that the lower release of cortical DA might influence frontal cognitive functions [82]. Synaptic NA in the PFC also plays a key role in attention, working memory, behavioral flexibility and response inhibition [83, 84]; all of them cognitive processes that exhibit altered patterns in schizophrenia. In contrast to DA, the status of the noradrenergic transmission in brain of schizophrenia patients has been scarcely investigated [85]. In fact, in vivo synaptic NA regulation in humans is unknown, probably due to absence of suitable radiotracers, whereas *postmortem* adrenoceptor expression and functionality in PFC of schizophrenia subjects remains unaltered [86]. Considering the relevance of the PFC catecholaminergic function in the pathophysiology of schizophrenia, the present study carried out a neurochemical characterization of the dopaminergic and noradrenergic activity in the PFC of offspring born of poly(I:C)-treated dams. Concretely, DA and NA synthesis, release, and reuptake processes, along with receptor availability, were evaluated in this specific MIA mouse model. Basal synaptic DA and NA concentrations were obtained by non-net flux microdialysis studies. In the present study, poly(I:C) mice presented a significant reduction of basal DA concentrations in PFC compared to controls, whereas extracellular basal NA values, although also lower, were not statistically significant. These results are compatible with a presynaptic dopaminergic deficiency in the PFC of the poly(I:C) model. In agreement with this hypothesis, it has been described in rats that poly(I:C) offspring exhibits a reduction in both the number and the firing rate of spontaneously active DA cells together with an altered activity pattern of the ventral tegmental area (VTA) [74, 87, 88].

In order to delve into the origin of reduced synaptic DA concentrations in poly(I:C) mice, action potential-dependent (K^+^ infusion) and action potential-independent (amphetamine administration) neurotransmitter release were evaluated in vivo. Expression of the presynaptic catecholamine synthesis-limiting enzyme TH and the reuptake transporters DAT and NET, together with functional neurotransmitter extraction fractions (Ed) were also analyzed. In this regard, a significant delay in the maximal effect of hyperK^+^ was observed for both DA and NA in the poly(I:C) group. Furthermore, the release of DA and NA induced by amphetamine was significantly lower in poly(I:C) mice compared to saline mice. These two results provide evidence for impairment in the mechanisms involved in catecholamine release from terminals. Earlier studies have also evaluated the stimulating effects of amphetamine in different MIA models of mice [51, 75, 89] and rats [90–92]. In general, rodent offspring born of poly(I:C)-injected dams exhibits enhanced locomotor activity in response to amphetamine, which is considered as an endophenotype of positive schizophrenia-like symptoms. Locomotor responsivity to amphetamine is driven by DA release in mesolimbic pathways [93, 94]. Therefore, it is widely accepted that underlying the increased locomotor responses to amphetamine in MIA offspring is a higher DA release in striatal areas. This striatal hyperdopaminergic state overlaps with the cortical hypodopaminergia obtained here and both mirror findings in schizophrenia patients [14].

Cortical NMDA receptor hypofunction is at the center of much current research on schizophrenia pathogenesis. It has long been known that acute pharmacological blockade of NMDA receptors worsens psychosis in schizophrenia patients and induces not only positive, but also schizophrenia cognitive and negative symptoms in healthy subjects [95, 96]. Consistently with previous studies, MK-801 increased DA and NA in the PFC [97–103]. The specific neurochemical mechanism by which NMDA receptor blockade regulates dopaminergic and noradrenergic activity and neurotransmitter release in the PFC is not yet fully understood [104]. It has been suggested that NMDA receptors located in GABAergic interneurons in the PFC are under tonic activation of endogenous glutamate [105] and that blocking this subpopulation of NMDA receptors increases the activity of glutamatergic downstream pathways [106]. Analogous mechanisms have been proposed for PFC control of the locus coeruleus noradrenergic area, although it has not yet been sufficiently demonstrated [107]. Results indicated that MK-801 induced similar DA and NA increases between groups, discarding the idea that poly(I:C) animals display impaired catecholamine release due to PFC dysfunctional NMDA receptors. Overall, all the different microdialysis release experiments link poly(I:C)-induced MIA with presynaptic cortical catecholaminergic abnormalities that become evident in adulthood. Previous results have already shown that this prenatal immune challenge may induce multiple defects in the development of the fetal DA system that will compromise the integrity of this neurotransmission system in the adulthood [89]. Therefore, dopaminergic and perhaps noradrenergic pathways emerge as critical primary targets of neurodevelopment alterations subsequent to viral-like immune activation by poly(I:C), contributing to disrupted behaviours and brain abnormalities that resemble schizophrenia phenotypes.

The possible relationship between presynaptic disturbances observed in the MIA model and alterations of TH was explored. There were no differences between poly(I:C) mice and controls in frontal cortex TH immunodensity. In accordance, no differences in DA and NA tissue contents in the whole cortex, the functional consequences of TH activity, were observed. These observations argue the idea that catecholamine synthesis in brain cortex is preserved and are concordant with previous studies where no differences were described either when measuring PFC DA [75, 108] or NA tissue concentrations [109] in the MIA model. However, discrepancies can be found in the literature where increased immunoexpression of TH has been reported in the PFC of prepuberal MIA mice originated from MIA by a higher and delayed poly(I:C) dose (20 mg/kg at GD12) [110].

In the same way, no differences in DAT or NET protein expression were observed between groups. Complementary, Ed obtained from non-net flux microdialysis displayed values for DA and NA that were not different between poly(I:C) and controls, supporting the idea that not only the expression, but also the function of catecholamine transporters, seems to be intact.

Finally, the potential disruption of dopaminergic signaling was also assessed by measuring protein expression of DA receptors. Human and animal studies have described that D_1_R and D_2_Rs are expressed in the PFC [111, 112], with a more prominent expression of D_1_Rs over D_2_Rs [113–115]. D_1_R is mainly postsynaptic [111] while D_2_R has been reported to be localized both pre-and post-synaptically, and in extra- and peri-synaptic structures of the PFC [116, 117]. In poly(I:C) mice, increased immunodensity was observed for both D_1_Rs and D_2_Rs, showing a positive correlation between them. These data are consistent with the existence of a PFC presynaptic hypodopaminergia that, following sustained DA deficit, would lead to compensatory up-regulation of postsynaptic D_1_Rs and D_2_Rs in the area. In fact, chronic DA depletion in rodents is associated with increased in vivo PFC [^11^C]NNC 112 binding, which presumably reflects a compensatory upregulation of D_1_R, influenced by changes in endogenous DA tone [118]. The present findings are congruent with reports describing that schizophrenia patients show higher D_1_R receptor availability than controls, particularly in dorsolateral PFC [119]. Although with a different dose of poly(I:C) (20 mg/kg) and prenatal timing (GD12), findings in the present work agree with studies where increased D_2_R mRNA and protein expression in the PFC of poly(I:C) mice were described [110]. Other authors have reported reduced immunohistochemical density of D_1_Rs and D_2_Rs in PFC of adult poly(I:C) male but not female animals [51]. One limitation of the present study is the lack of information in female animals. Some poly(I:C)-based studies have shown sex-specific effects of MIA with predominant male vulnerability at several time points after exposition [34]. Therefore, a potential disparity between male and female in relation to the cortical hypodopaminergia phenotype reported here deserves further investigation.

## Conclusions

As summary, the findings highlight that prenatal exposure to the immunoactive agent poly(I:C) can negatively affect the normal development of the mesocortical dopaminergic pathway. In adulthood, the MIA poly(I:C) animal model exhibits in vivo impairment of the DA- and NA-evoked release from PFC nerve terminals leading to reduced neurotransmitter availability in the synapsis, and accompanied by compensatory upregulation of DA receptors. Taken together with the existing evidence of enhanced mesolimbic activity, the herein characterized poly(I:C)-based MIA model embraces one of the main phenotypes reported in schizophrenia, i.e., the coexistence of striatal hyper-dopaminergia with prefrontal hypo-dopaminergia. In consequence, this animal model represents an outstanding opportunity for the study of cognitive impairment associated to schizophrenia and the rational development of new pro-cognitive strategies.

### Electronic Supplementary Material

Below is the link to the electronic supplementary material.

**Additional file 1. Supplementary table 1**: Summary table of animals intended for each experimental technique and the number of treated females they were originated from. “Treated dams” columns indicate the number of females administrated with poly(I:C)/saline. “Offspring” columns indicate the total number of pups from those females. “Used males” columns indicate the number of males used and represented in results for each experimental technique.

**Additional file 2**

**Additional file 3. Supplementary Fig. 1Representative Western blot images of the expression of different proteins in brain cortex.** Expression of three saline control and three poly(I:C) mice are shown. Images represent immunoblots of: **(A)** DAT, **(B)** NET, **(C)** TH (with Santa Cruz antibody), **(D)** TH (with Abcam antibody), **(E)** dopamine D_1_ receptor and **(F)** dopamine D_2_ receptor. Each protein blot is accompanied by the respective β-actin blot.

**Additional file 4. Supplementary Fig. 2Novel object exploration test.** Representation of **(A)** the exploration time (in seconds) devoted to the familiar object; **(B)** the exploration time (in seconds) devoted to the novel object and **(C)** the total exploration time (in seconds) of both familiar and novel objects by the saline control (n = 9) and the poly(I:C) mice (n = 15) during the NORT. Bars represent mean ± SEM values.

**Additional file 5: Supplementary Fig. 3** Exploration time Devoted to the Familiar Object and to the Novel Object Representation of the familiar and novel exploration time (in seconds) of saline control (n = 9) and poly(I:C) (n = 15) mice. Bars represent mean ± SEM values. ***p < 0.0001, two-way ANOVA followed by Bonferroni post-hoc test. ns: non significant.

**Additional file 6. Supplementary Fig. 4Catecholamine tissue concentrations.** Concentrations (nM/mg fresh tissue) of **(A)** DA and **(B)** NA in brain cortex of saline control (n = 21) and poly(I:C) (n = 19–20) mice. Bars represent mean ± SEM values.

**Additional file 7. Supplementary Fig. 5** Correlations Between Different Targets of Catecholamine Neurotransmission Systems. A) Correlation between the TH expression evaluated by the Santa Cruz and the Abcam TH antibodies (r = 0.92, n = 49, p < 0.0001); **B)** Correlation between the TH expression (Santa Cruz antibody) and the DAT expression (r = 0.63, n = 49, p < 0.0001); **C)** Correlation between the TH expression (Abcam antibody) and the DAT expression (r = 0.68, n = 49, p < 0.0001); **D)** Correlation between the D_1_R and D_2_R expression (r = 0.74, n = 50, p < 0.0001).

## Data Availability

The datasets used and/or analysed during the current study are available from the corresponding author on reasonable request.

## Abbreviations

CIAS: Cognitive impairment associated with schizophrenia
COMT: Catechol-o-methyltransferase
DA: Dopamine
D_1_R: Dopamine D_1_ receptor
D_2_R: Dopamine D_2_ receptor
DAT: Dopamine transporter
Ed: Extraction fraction
GD: Gestational day
MIA: Maternal immune activation
NA: Noradrenaline
NET: Noradrenaline transporter
NMDA: N-methyl-D-Aspartate
NORT: Novel object recognition test
OCT: Organic cation transporter
PCP: Phencyclidine
PET: Positron emission tomography
PMAT: Plasma membrane monoamine transporter
Poly(I:C): Polyriboinosinic-polyribocytidylic acid
PD: Postnatal day
PFC: Prefrontal cortex
TLR3: Toll-like receptor 3
TH: Tyrosine hydroxylase
