# Supplementary material for: Maternal Immune Activation Induces Cortical Catecholaminergic Hypofunction and Cognitive Impairments in Offspring
Source: J Neuroimmune Pharmacol. 2023 May 20;18(3):348–65. doi: 10.1007/s11481-023-10070-1 (PMC10577104; doi:10.1007/s11481-023-10070-1)
Supplement: Supplementary file 1 — Additional file 1. Supplementary table 1: Summary table of animals intended for each experimental technique and the number of treated females they were originated from. “Treated dams” columns indicate the number of females administrated with poly(I:C)/saline. “Offspring” columns indicate the total number of pups from those females. “Used males” columns indicate the number of males used and represented in results for each experimental technique. [file 11481_2023_10070_MOESM1_ESM.docx]

**Additional file 1. Supplementary table 1:** Summary table of animals intended for each experimental technique and the number of treated females they were originated from. “Treated dams” columns indicate the number of females administrated with poly(I:C)/saline. “Offspring” columns indicate the total number of pups from those females. “Used males” columns indicate the number of males used and represented in results for each experimental technique.

|  | Poly(I:C) | | | | |  | Saline | | | | |
| --- | --- | --- | --- | --- | --- | --- | --- | --- | --- | --- | --- |
|  | Treated dams |  | Offspring |  | Used males |  | Treated dams |  | Offspring |  | Used males |
| NORT | 4 |  | 27 |  | 15 |  | 3 |  | 26 |  | 9 |
| Microdialysis | 28 |  | 120 |  | 51 |  | 26 |  | 114 |  | 50 |
| Tissue monoamines | 12 |  | 32 |  | 20 |  | 10 |  | 45 |  | 21 |
| Western blot | 12 |  | 56 |  | 27 |  | 11 |  | 53 |  | 23 |
