# Supplementary material for: Maternal Immune Activation Induces Cortical Catecholaminergic Hypofunction and Cognitive Impairments in Offspring
Source: J Neuroimmune Pharmacol. 2023 May 20;18(3):348–65. doi: 10.1007/s11481-023-10070-1 (PMC10577104; doi:10.1007/s11481-023-10070-1)
Supplement: Supplementary file 2 — Additional file 2 [file 11481_2023_10070_MOESM2_ESM.docx]

**Selection of antibodies**

Since the reliability of Western blot is determined by the specificity of the antibodies, an important effort was made in their election. We selected, whenever possible, antibodies that had been validated using knock-out (KO)-mice. DAT antibody was validated by our team using DAT-KO mice [1], and in these experiments showed a band migrating at 70-80 kDa (**Additional file 3: Supplementary Fig. 1A**). NET antibody has also been previously characterized in KO-mice for this transporter [2], and visualized the protein at approximately 70 kDa (**Additional file 3: Supplementary Fig. 1B**). Regarding TH, since we did not find any antibody validated in KO-mice, two different antibodies were chosen, both having been largely used and published as dopaminergic neuronal markers. The Santa Cruz antibody (SC25269) against TH was selected because its specificity had previously been reported in another MIA mouse model [3]. As for the Abcam antibody (AB41528), we validated it with the blocking peptide, which completely erased the signal (data not shown). In both cases, we quantified the most intense band migrating at 55-60 kDa, which corresponds to the predicted molecular weight (**Additional file 3: Supplementary Fig. 1C and 1D**). The specificity of the D_1_R antibody had also been previously verified in striatal D_1_R KO-mice tissue [4], and showed the predicted profile with a very intense band at 50 kDa (**Additional file 3: Supplementary Fig. 1E**). Regarding D_2_R antibody, its specificity was validated in cultured cells that do not express D_2_Rs [5], and we confirmed that the protein was visualized at the predicted molecular weight of 50 kDa (**Additional file 3: Supplementary Fig. 1F**). For both D_1_R and D_2_R blots, other fainter bands were also visible at 60-65 kDa, but since their exact identity remains unknown, they were not quantified.

**References**

1. Erdozain AM, De Gois S, Bernard V, Gorgievski V, Pietrancosta N, Dumas S, et al. Structural and Functional Characterization of the Interaction of Snapin with the Dopamine Transporter: Differential Modulation of Psychostimulant Actions. Neuropsychopharmacology. 2018;43(5):1041-51.

2. Matthies HJ, Han Q, Shields A, Moore JL, Winder DG, Galli A, et al. Subcellular localization of the antidepressant-sensitive norepinephrine transporter. BMC Neurosci. 2009;10:65.

3. Deslauriers J, Larouche A, Sarret P, Grignon S. Combination of prenatal immune challenge and restraint stress affects prepulse inhibition and dopaminergic/GABAergic markers. Prog Neuropsychopharmacol Biol Psychiatry. 2013;45:156-64

4. Keeler BE, Lallemand P, Patel MM, de Castro Brás LE, Clemens S. Opposing aging-related shift of excitatory dopamine D1 and inhibitory D3 receptor protein expression in striatum and spinal cord. J Neurophysiol. 2016;115(1):363-69.

5. Luessen DJ, Hinshaw TP, Sun H, Howlett AC, Marrs G, McCool BA, et al. RGS2 modulates the activity and internalization of dopamine D2 receptors in neuroblastoma N2A cells. Neuropharmacology. 2016;110(Pt A):297-307.
