# Supplementary figures and images for: Maternal Immune Activation Induces Cortical Catecholaminergic Hypofunction and Cognitive Impairments in Offspring
Source: J Neuroimmune Pharmacol. 2023 May 20;18(3):348–65. doi: 10.1007/s11481-023-10070-1 (PMC10577104; doi:10.1007/s11481-023-10070-1)

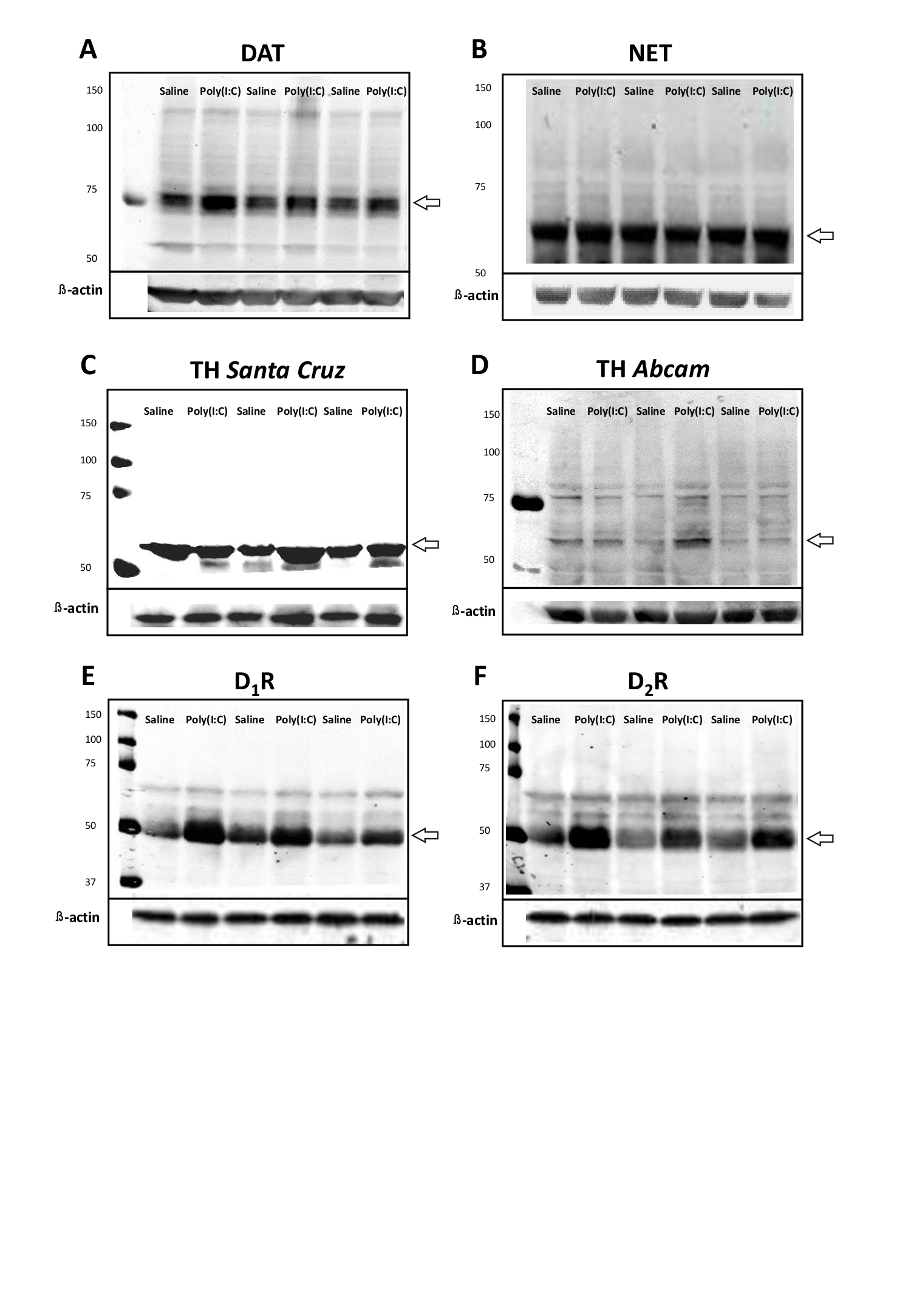

Supplement: Supplementary file 3 — Additional file 3. Supplementary Fig. 1Representative Western blot images of the expression of different proteins in brain cortex. Expression of three saline control and three poly(I:C) mice are shown. Images represent immunoblots of: (A) DAT, (B) NET, (C) TH (with Santa Cruz antibody), (D) TH (with Abcam antibody), (E) dopamine D1 receptor and (F) dopamine D2 receptor. Each protein blot is accompanied by the respective β-actin blot. [file 11481_2023_10070_MOESM3_ESM.tiff]

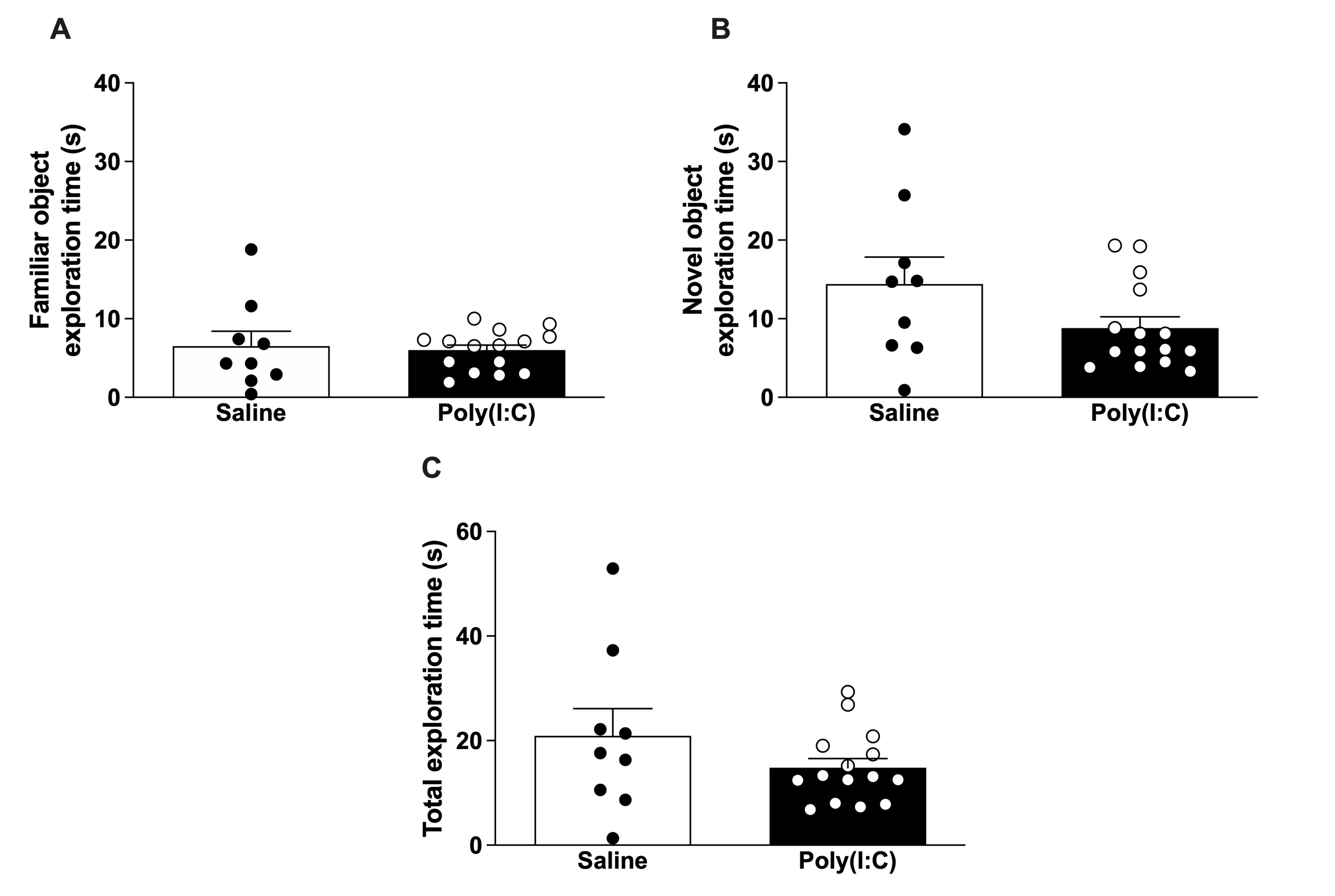

Supplement: Supplementary file 4 — Additional file 4. Supplementary Fig. 2Novel object exploration test. Representation of (A) the exploration time (in seconds) devoted to the familiar object; (B) the exploration time (in seconds) devoted to the novel object and (C) the total exploration time (in seconds) of both familiar and novel objects by the saline control (n = 9) and the poly(I:C) mice (n = 15) during the NORT. Bars represent mean ± SEM values. [file 11481_2023_10070_MOESM4_ESM.tiff]

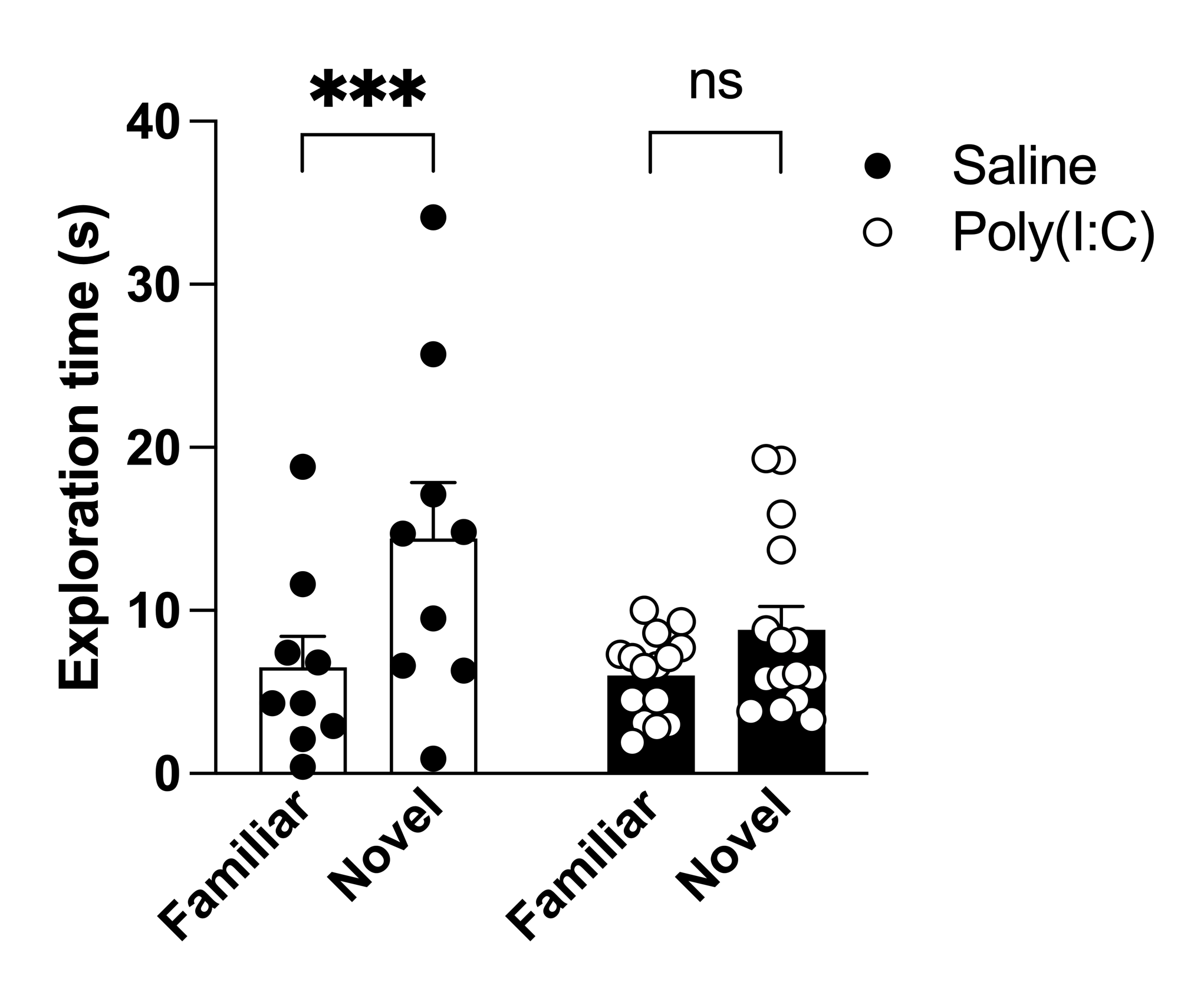

Supplement: Supplementary file 5 — Additional file 5: Supplementary Fig. 3 Exploration time Devoted to the Familiar Object and to the Novel Object Representation of the familiar and novel exploration time (in seconds) of saline control (n = 9) and poly(I:C) (n = 15) mice. Bars represent mean ± SEM values. ***p < 0.0001, two-way ANOVA followed by Bonferroni post-hoc test. ns: non significant. [file 11481_2023_10070_MOESM5_ESM.tiff]

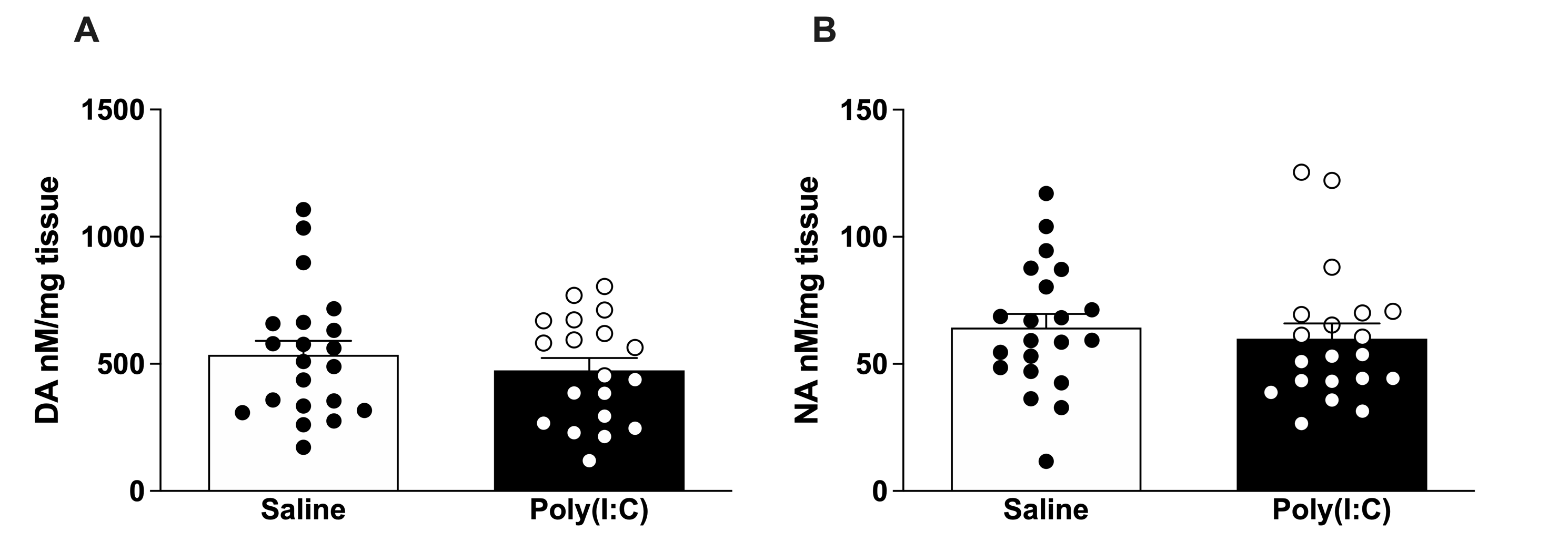

Supplement: Supplementary file 6 — Additional file 6. Supplementary Fig. 4Catecholamine tissue concentrations. Concentrations (nM/mg fresh tissue) of (A) DA and (B) NA in brain cortex of saline control (n = 21) and poly(I:C) (n = 19–20) mice. Bars represent mean ± SEM values. [file 11481_2023_10070_MOESM6_ESM.tiff]

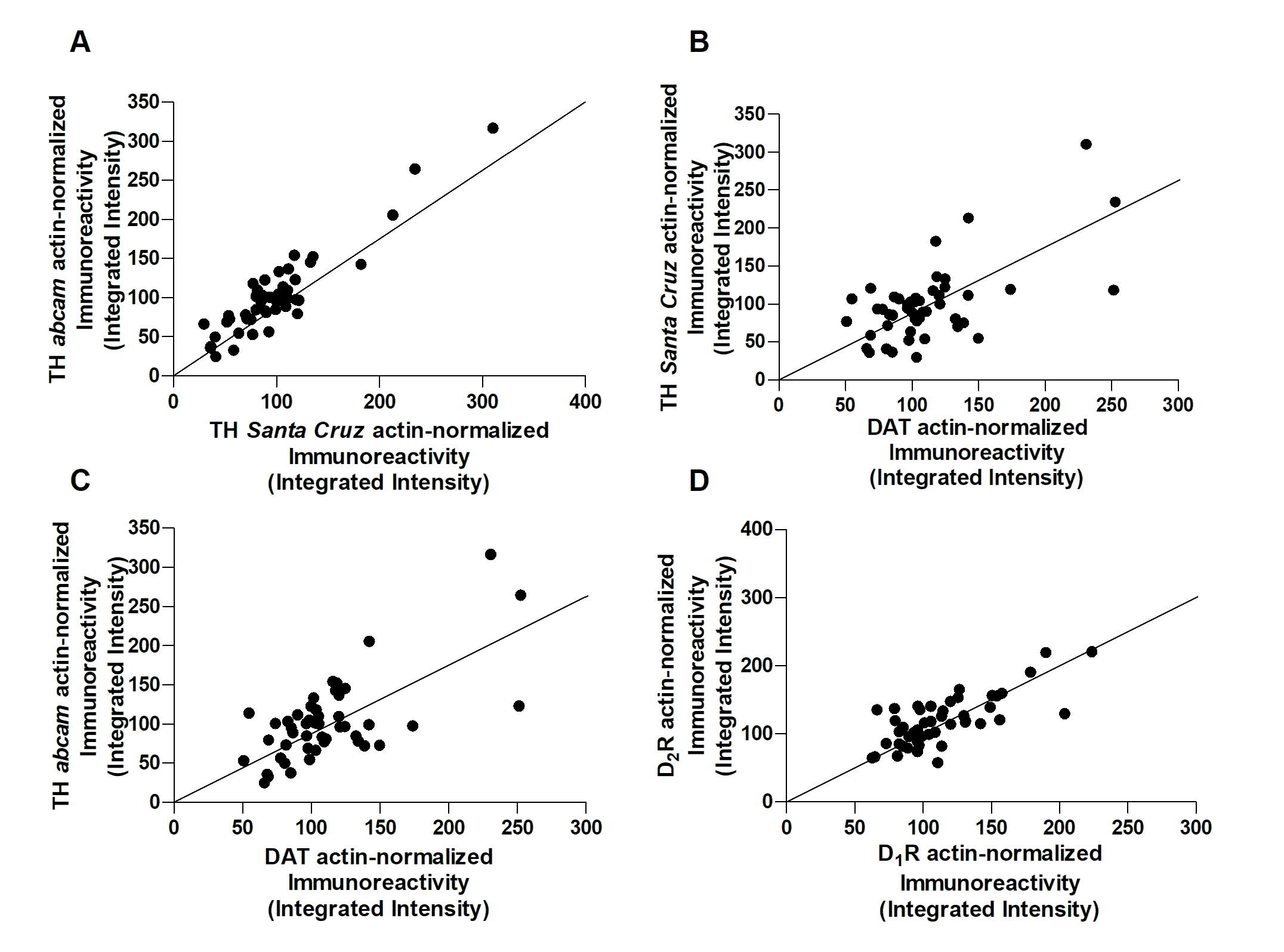

Supplement: Supplementary file 7 — Additional file 7. Supplementary Fig. 5 Correlations Between Different Targets of Catecholamine Neurotransmission Systems. A) Correlation between the TH expression evaluated by the Santa Cruz and the Abcam TH antibodies (r = 0.92, n = 49, p < 0.0001); B) Correlation between the TH expression (Santa Cruz antibody) and the DAT expression (r = 0.63, n = 49, p < 0.0001); C) Correlation between the TH expression (Abcam antibody) and the DAT expression (r = 0.68, n = 49, p < 0.0001); D) Correlation between the D1R and D2R expression (r = 0.74, n = 50, p < 0.0001). [file 11481_2023_10070_MOESM7_ESM.tif]
